# Transcriptome profiling confirmed correlations between symptoms and transcriptional changes in RDV infected rice and revealed nucleolus as a possible target of RDV manipulation

**DOI:** 10.1186/1743-422X-11-81

**Published:** 2014-05-06

**Authors:** Liang Yang, Zhenguo Du, Feng Gao, Kangcheng Wu, Lianhui Xie, Yi Li, Zujian Wu, Jianguo Wu

**Affiliations:** 1 Key Laboratory of Plant Virology of Fujian Province, Institute of Plant Virology, Fujian Agriculture and Forestry University, Fuzhou, Fujian 350002, China; 2 Key Laboratory of Biopesticide and Chemibiology of Ministry of Education, Fujian Agriculture and Forestry University, Fuzhou, Fujian 350002, China; 3 Peking-Yale Joint Center for Plant Molecular Genetics and Agrobiotechnology, The State Key Laboratory of Protein and Plant Gene Research, College of Life Sciences, Peking University, Beijing 100871, China; 4 Guangdong Provincial Key Laboratory of High Technology for Plant Protection, Guangzhou 510640, China

**Keywords:** RDV, Transcriptome profiling, Pns11, Nucleolus

## Abstract

**Background:**

*Rice dwarf virus* (RDV) is the causal agent of rice dwarf disease, which limits rice production in many areas of south East Asia. Transcriptional changes of rice in response to RDV infection have been characterized by Shimizu et al. and Satoh et al.. Both studies found induction of defense related genes and correlations between transcriptional changes and symptom development in RDV-infected rice. However, the same rice cultivar, namely *Nipponbare* belonging to the *Japonic* subspecies of rice was used in both studies.

**Methods:**

Gene expression changes of the *indica* subspecies of rice, namely *Oryza sativa* L. ssp. *indica* cv Yixiang2292 that show moderate resistance to RDV, in response to RDV infection were characterized using an Affymetrix Rice Genome Array. Differentially expressed genes (DEGs) were classified according to their Gene Ontology (GO) annotation. The effects of transient expression of Pns11 in *Nicotiana benthaminana* on the expression of nucleolar genes were studied using real-time PCR (RT-PCR).

**Results:**

856 genes involved in defense or other physiological processes were identified to be DEGs, most of which showed up-regulation. Ribosome- and nucleolus related genes were significantly enriched in the DEGs. Representative genes related to nucleolar function exhibited altered expression in *N. benthaminana* plants transiently expressing Pns11 of RDV.

**Conclusions:**

Induction of defense related genes is common for rice infected with RDV. There is a co-relation between symptom severity and transcriptional alteration in RDV infected rice. Besides ribosome, RDV may also target nucleolus to manipulate the translation machinery of rice. Given the tight links between nucleolus and ribosome, it is intriguing to speculate that RDV may enhance expression of ribosomal genes by targeting nucleolus through Pns11.

## Background

Viruses are obligate intracellular pathogens. They hijack host functions, divert host resources and suppress host defense responses to achieve successful infection [1]. These involve an array of interactions with cellular factors, which, inevitably or coincidentally, often lead to host physiological disorders manifested by a variety of disease symptoms [2,3]. Understanding molecular details from infection of a virus to symptom development of the host is one major mission of plant virologists. Transcriptome profiling has been used extensively in the past decade to understand mechanisms underlying plant-virus interaction [4,5]. Transcriptional response of plants to virus infection is shown to vary depending on virus species, virus strains and the genetic backgrounds of host plants [6-8]. However, it shows a tight link with phenotypes and thus is useful to reveal how a virus colonizes a host, how a host mounts a defense response against a virus, and how a compatible virus-host interaction results in disease symptoms [6-8]. Also, these studies find that some genes may be commonly regulated by different viruses in different host plants [9]. For example, a set of ribosomal genes have been shown to be up-regulated in Arabidopsis, *Nicotiana benthamiana* and rice infected with *Turnip mosaic virus* (TuMV), Plum pox potyvirus (PPV) and *Rice stripe virus* (RSV), respectively [10-12].

Rice, one of the main crop plants as well as a model for monocot plant research [13], is host to many viruses. Among them, *Rice dwarf virus* (RDV), a member of the genus *Phytoreovirus* in the family *Reoviridae*, is one of the most widespread and disastrous rice-infecting viruses causing great yield reduction in south East Asia [14-16]. RDV is transmitted in a propagative and circulative manner by leafhoppers (*Nephotettix spp.*) [17]. Typical symptoms associated with RDV infection include severe dwarfism, increased tilling and white chlorotic specks on the infected leaves [18].

RDV are icosahedral double-shelled particles of approximately 70 nm in diameter. The genome of RDV is composed of 12 segments of double stranded RNAs, which are named S1-S12, respectively, according to their migration during sodium dodecyl sulfate–polyacrylamide gel electrophoresis. S1, S2, S3, S5, S7, S8, and S9 encode seven structural proteins, namely, P1, P2, P3, P5, P7, P8, and P9, respectively. P1, a putative RNA polymerase; P5, a putative guanylyltransferase; and P7, a nonspecific nucleic acid binding protein form the core of RDV together with viral dsRNAs [19]. P3 and P8 are major components of the inner and outer protein shells that encapsidate the core, respectively [20,21]. P2 and P9 are minor components of the outer capsid [22,23]. The structural features and the process of assembly of RDV virions have been well studied [24,25]. Besides structural proteins, RDV encodes at least five non-structural proteins, namely Pns4, Pns6, Pns10, Pns11, and Pns12, respectively. Pns6, Pns11 and Pns12 are matrix proteins of viroplasm, which is the putative site of viral replication [26]. Pns4 is a phosphoprotein and is localized around the viroplasm matrix in insect cells [27]. Several proteins of RDV have been shown to play specific roles in RDV-rice interaction. For example, Pns6 was identified as a viral movement protein and Pns10 as a RNA silencing suppressor of RDV [28,29]. P2 interacts with ent-kaurene oxidases of rice, which leads to reduced biosynthesis of gibberellins and rice dwarf symptoms [30].

In this study, the transcriptome of the *indica* subspecies of rice, namely *Oryza sativa* L. ssp. *indica* cv Yixiang2292, in response to RDV infection was profiled using Affymetrix GeneChips, which contains probes representing the entire genome of rice [13] (http://www.affymetrix.com). Our results further confirm the notion that induction of defense related genes is common for rice infected with RDV and there are correlations between transcriptional changes and symptom development in RDV-infected rice.

## Results

### Transcriptome profiling of RDV-infected rice

For transcriptome analysis, rice seedlings were virus- or mock- inoculated. Total RNAs were extracted at 22 days post inoculation (dpi), i.e. the earliest time when infection could be confirmed by the appearance of symptoms. The GeneChip hybridization and scanning were performed at the Microarray Resource Laboratory at Beijing CapitalBio Corporation, Beijing, China, in which GeneChip microarray service was certificated by Affymetrix. The microarray data were analyzed using SAM (Significant Analysis of Microarray) software. Deferentially expressed genes (DEGs) were identified with the criteria of fold changes > 1.5 and false positive rate (*q*-value) < 0.058. In this way, a total of 856 genes were identified to be DEGs, in which 838 genes were upregulated and 18 downregulated. A list of the genes identified is presented in Additional file 1: Table S1.

### Classification of DEGs

**Figure 1:**
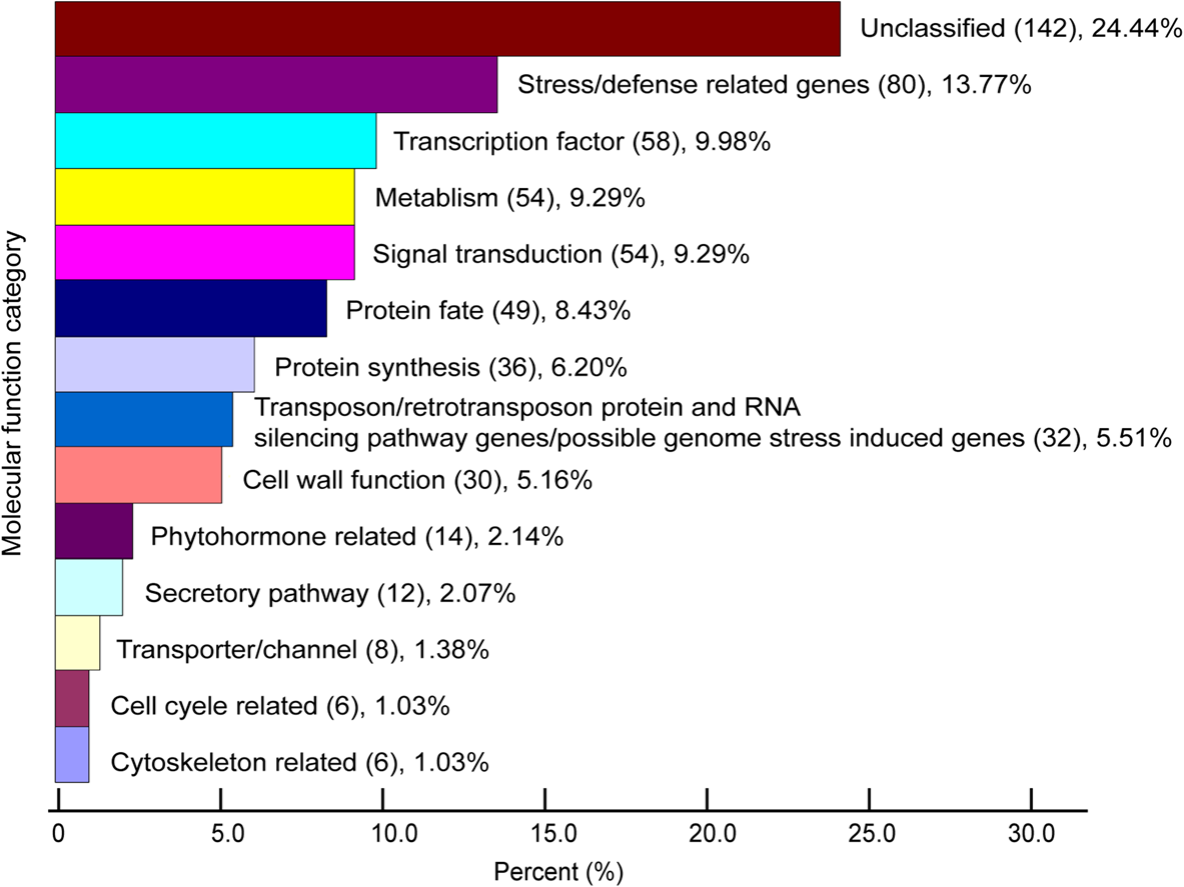
**An overview of the functional classification of the 581 RDV responsive genes.** Number of genes and relative percentage for each category were indicated. For a list of the genes in each category see Additional file 2: Table S2.

To get an overview of the functions of the DEGs, DEGs were classified according to their function. The classification was done manually based on gene annotations (http://rice.plantbiology.msu.edu/) and literature searching. Among the 856 DEGs, 275 genes have no annotations or were simply annotated as hypothetical protein/expressed protein. These genes were not analyzed further in our study. The remaining 581 genes were classified into 14 non-redundant categories (Figure 1, Additional file 2: Table S2). As shown in Figure 1, unclassified genes formed the largest group. They referred to genes that were difficult to be classified into groups. Of the genes that have been classified, three categories are of particular interests to us.

### Defense/stress related genes

This set of genes forms the second largest group. They include PR genes, markers of defense responses; several genes encoding WRKY transcription factors, key regulators of defense responses [31]; L-ascorbate oxidase and Peroxidase genes, important modulators of oxidative stress, among others [32].

### Protein synthesis related genes

In all, 36 RDV responsive genes were classified into this category (Figure 1). The large number of this category was due mostly to ribosomal genes. These included 27 genes encoding cytosolic ribosomal subunits, 2 mitochondrial ribosomal genes and 1 gene encoding a chloroplast ribosome precursor. Other genes belonging to this category include those involved in translation initiation, termination and tRNA metabolism. All these genes were up-regulated.

### Transposon/retrotransposon protein/RNA silencing pathway genes/possible genome stress related genes

Surprisingly, a large number of genes encoding transposon/retrotransposon-related proteins were affected (Figure 1). Normally, tansposon or transposon-related genes are transcriptional inert because of epigenetic regulations. Altered expression of these kinds of genes indicated that the rice genome was suffering a genomic stress. Indeed, genes involved in DNA recombination (AK063836, encoding a Single-strand binding protein family protein; BQ908269, encoding a RuvB-like 1 protein; AB079873, encoding a Meiotic recombination protein DMC1 homolog.), DNA repair (AK101485, encoding a DNA repair ATPase) and chromosome assembly (AK108572, encoding a complex 1 protein containing protein) were all upregulated. The RNA silencing pathway, which plays a pivotal role in epigenetic regulations, was also significantly affected. Genes functioning in this pathway such as *AGOs*, *RDRs* showed marked up-regulation (Figure 1, Additional file 2: Table S2).

### GO enrichment analysis

DEGs were also classified according to Gene Ontology (GO) cellular component, which indicates the location or suspected location of a gene in a cell [33]. As shown in Table 1, six GO cellular component terms were significantly enriched in DEGs, cell wall, nucleus, ribosome, cytosol, extracellular region, and nucleolus (*p* < 0.01). We were interested in the concomitant enrichment of the two GO terms Ribosome and Nucleolus, because nucleolus is the site of ribosomal RNA synthesis and ribosome maturation.

**Table 1:** Results of GO cellular component analysis with MAS 2.0 system

**GO number**	**Cellular component**	**Total change genes**	***p*-value**	***q*-value**
GO:0005618	Cell wall	112	0.0	0.0
GO:0005634	Nucleus	101	0.0	0.0
GO:0005840	Ribosome	24	0.0	0.0
GO:0005829	Cytosol	21	1.0E-6	3.0E-6
GO:0005576	Extracellular region	14	3.64E-4	5.24E-4
GO:0005730	Nucleolus	20	0.0014	0.0018
GO:0005886	Plasma membrane	23	0.0128	0.0148
GO:0016020	Membrane	103	0.0152	0.016
GO:0005740	Mitochondrial envelope	2	0.1273	0.1182
GO:0005794	Golgi apparatus	4	0.1314	0.1217
GO:0005635	Nuclear envelope	3	0.2492	0.2231
GO:0005856	Cytoskeleton	7	0.3283	0.2924
GO:0005783	Endoplasmic reticulum	2	0.3565	0.3161
GO:0016023	Cytoplasmic membrane-bound vesicle	62	0.4074	0.3457
GO:0005773	Vacuole	3	0.5308	0.4267
GO:0005654	Nucleoplasm	1	0.5373	0.4314
GO:0005777	Peroxisome	1	0.671	0.5287
GO:0009579	Thylakoid	12	0.6852	0.5375
GO:0005739	Mitochondrion	137	0.9989	0.5485
GO:0009536	Plastid	65	1.0	0.5485
GO:0005622	Intracellular	10	1.0	0.5485
GO:0005737	Cytoplasm	82	1.0	0.5485
GO:0005575	Cellular_component	3	1.0	0.5485
GO:0005623	Cell	8	1.0	0.5485

### Verification of the microarray data

**Figure 2:**
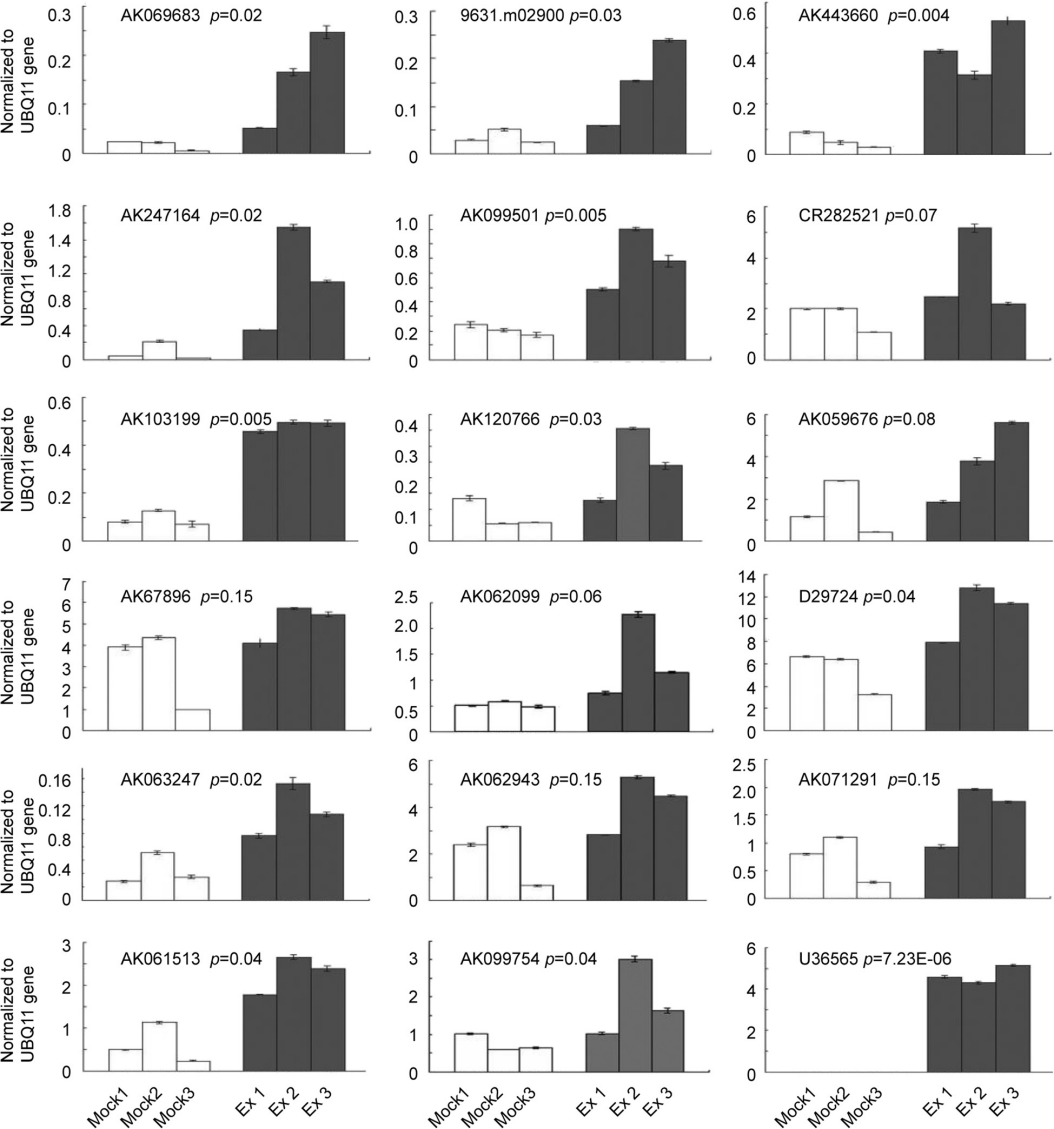
**Validation of microarray results using qRT-PCR.** Shown are relative expression ratios to UBQ11 in inoculated (gray bars (Ex 1, 2 and 3)) and mock-inoculated (Open bars (Mock 1, 2 and 3)) rice. Means of three replicate experiments with standard deviations and p-values are shown in Table 1.

The accuracy of the microarray data was verified by qRT-PCR. Seventeen genes including ribosomal, nucleolar and transposon/retrotransposon related genes and genes involved in RNA silencing, auxin signal, and cell wall function were selected. The CP gene of RDV was used to as a control. As shown in Figure 2 and Table 2, qRT-PCR results of all 17 RDV responsive genes selected were consistent with the microarray data.

**Table 2:** **Real-time PCR to verify expression pattern of differentially expressed genes from the microarray experiment (for a list of these genes primer sequence, see Additional file **2**: Table S2)**

**GB.accession**	**Fold change (Ex/Mock) ± SD**		***p*-value**^**B**^	***p*-value**^**A**^	**Description**
	**Microarray**	**qRT-PCR**			
AK069685	5.4983 ± 1.581	6.6246 ± 3.6546	0	0.0204	Piwi domain containing protein, expressed
9631.m02900	2.6141 ± 0.7001	2.9988 ± 0.530	0	0.0316	Small nuclear ribonucleoprotein G, putative, expressed
AF443600	6.2009 ± 2.7810	6.5142 ± 2.1441	0.0042	0.0045	Glucan endo-1,3-beta-glucosidase GII precursor, putative, expressed
AF247164	6.1081 ± 6.8408	6.7585 ± 11.4824	0.0236	0.0235	Alpha-expansin 4 precursor, putative, expressed
AK099501	2.1007 ± 0.9030	2.4711 ± 0.5329	0.0261	0.005	Ribonucleoprotein, putative, expressed
CR282531	1.7015 ± 0.2375	1.4174 ± 0.5866	0.0303	0.0694	60S acidic ribosomal protein P2A, putative, expressed
AK103199	3.2899 ± 2.5965	4.0319 ± 3.4615	0.0302	0.0057	Transposon protein, putative, CACTA, En/Spm sub-class, expressed
AK062099	1.6257 ± 0.3806	1.7461 ± 0.3890	0.0308	0.0601	Ribosomal L28e protein family protein, expressed
AK059679	1.8421 ± 0.303	1.9076 ± 0.9539	0.0308	0.0892	60S ribosomal protein L38, putative, expressed
AK067896	1.6171 ± 0.2825	1.3415 ± 0.6274	0.04342	0.1508	60S ribosomal protein L6, putative, expressed
AK120766	1.7459 ± 0.5397	2.1115 ± 0.8514	0.04342	0.0293	Piwi domain containing protein, expressed
D29724	1.529 ± 0.2032	1.51084 ± 0.2562	0.04342	0.0408	Peptide chain release factor 2, putative, expressed, 60S ribosomal protein L38, putative, expressed
AK063247	2.5979 ± 1.4057	2.0925 ± 2.0497	0.04342	0.0197	Auxin-induced protein TGSAUR12, putative, expressed
AK062943	1.5919 ± 0.0932	1.5679 ± 1.0226	0.0483	0.0986	40S ribosomal protein S15a, putative, expressed
AK071291	1.6782 ± 0.3353	1.5959 ± 0.6893	0.0483	0.0831	Fibrillarin-2, putative, expressed
AK061513	2.5152 ± 0.6761	2.8184 ± 1.9757	0.0588	0.0434	Nucleoid DNA-binding protein cnd41, putative, expressed
AK099754	1.7647 ± 0.2432	1.7471 ± 0.2758	0.0588	0.0487	Nucleolar protein NOP5, putative, expressed
U36565	–	4.8787 ± 2.0832	–	7.23E-06	Rice dwarf virus coat protein mRNA, complete cds
UBQ11*	–	–	–	0.1955	

Notice: ^A^*p*-value form microarray experiment; ^B^*p*-value from qRT-PCR; *Rice *UBQ11* was used as a control for qRT-PCR.

### The nucleoli were affected in RDV-infected rice

**Figure 3:**
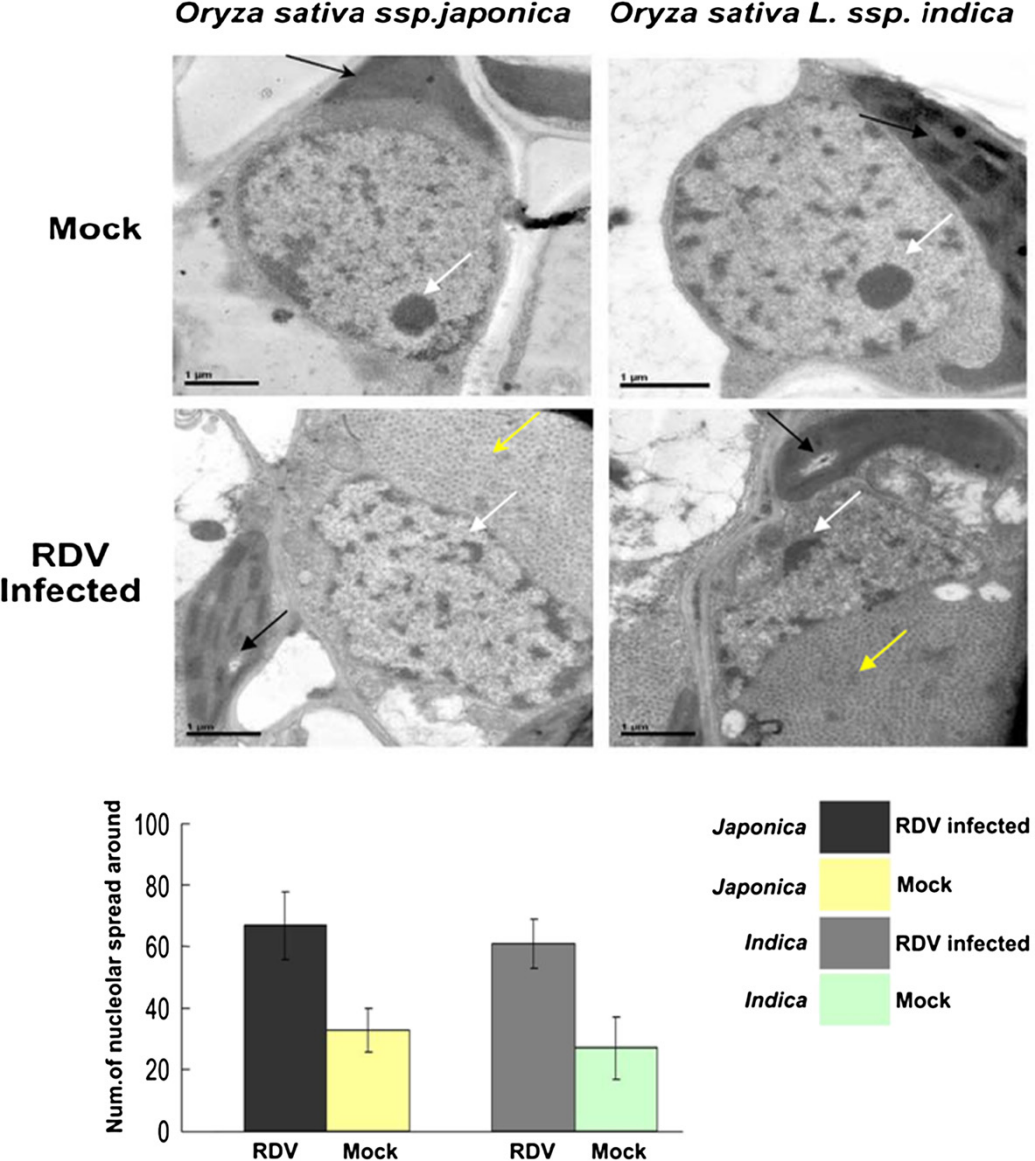
**The structure of nucleolus in RDV-infected rice.** White arrowhead: Nucleolus; Black arrowhead: Chloroplast; Yellow arrowhead: RDV Virion. Rectangle: the biological statistics of number of nucleolar spread around

Transmission electron microscopy was used to determine if there are any pathologic changes related to nucleoli in RDV-infected cells. As shown in Figure 3, two forms of nucleoli were observed in infected or control rice plants: small, round and concentrated electron-dense spheres (type 1) and big, irregular sub-cellular compartments filled with dispersive electron-dense aggregates (type 2). Statistical analysis confirmed that the number of type 2 nucleolus in RDV infected rice (61% ~ 67%) was higher than that of type 1, whereas in control rice plants, the number of type 1 nucleolus was higher than that of type 2 (27% ~ 33%).

### RDV Pns11 regulates the transcript levels of nucleolus-related genes in tobacco cells

**Figure 4:**
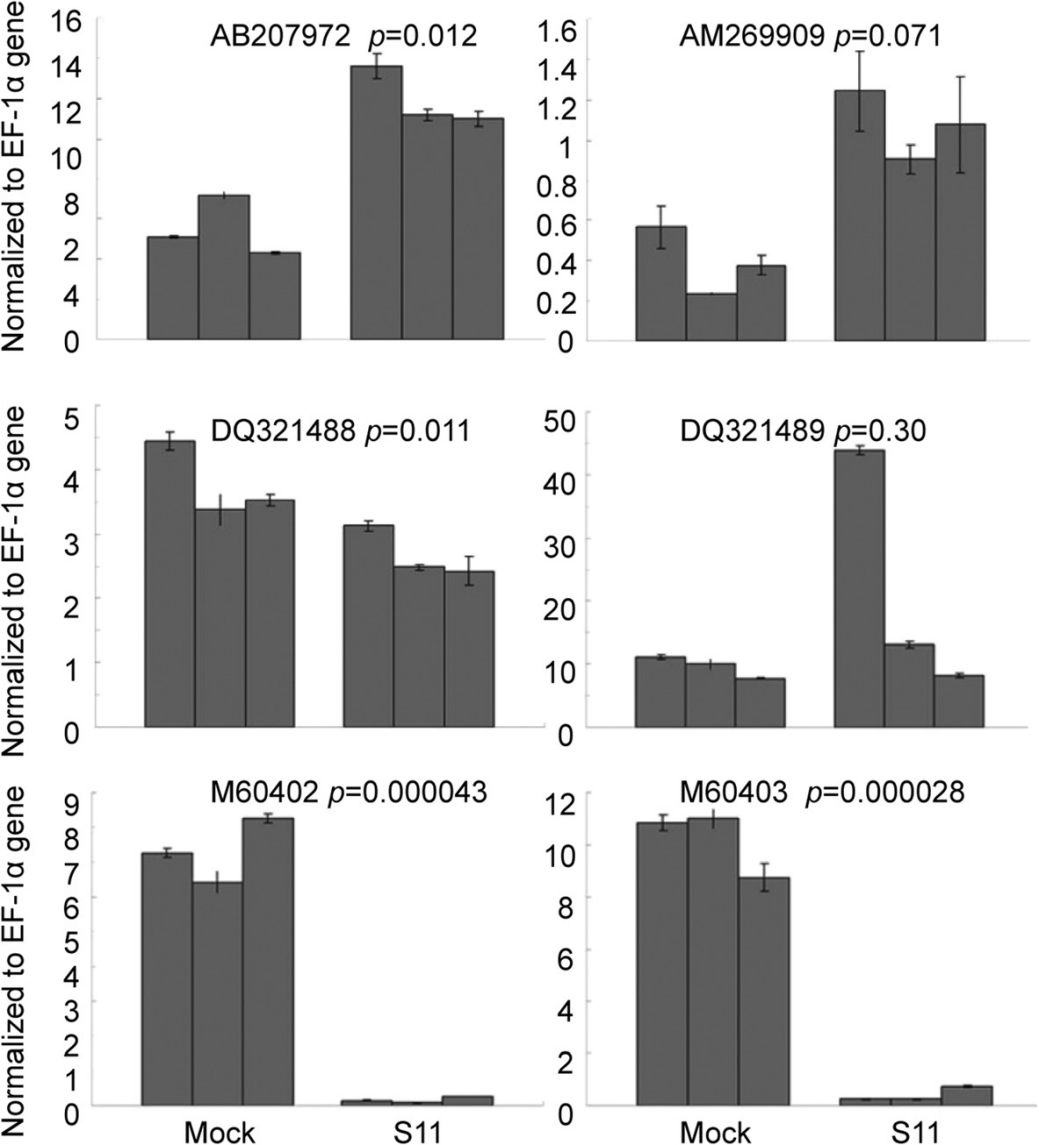
**RDV Pns11 regulates the transcript levels of nucleolus-related genes. (A)** Schematic representation of Nuclear localization signal (NLS) of RDV Pns11. **(B)** qRT-PCR was used to monitor the mRNA of nucleolus-related genes. Shown are relative expression ratios to EF-1α. Three replicate experiments were done to reduce biological variation.

The finding that many nucleolus targeting genes were de-regulated in RDV infected rice suggests that RDV may manipulate nucleolar functions. The subcellular localization of all RDV-encoded proteins was predicted by Predict NLS (http://www.biologydir.com/nls-prediction/p1.html). Only Pns11 has a nuclear localization signal (NLS) with NLSIand NLSIIdomains belonging to the bipartite NLS [34] (Figure 4A). So Pns11 may be responsible for alteration of nucleolar genes in RDV-infected rice. To test this possibility, the expression levels of two nucleolar genes were studied in *N. benthamiana* leaves expressing RDV Pns11. qRT-PCR results revealed that the two genes (AB207972 and AM269909 encoding fibrillarin) were upregulated significantly. As controls, two genes related to defense (Glucan endo-1,3-beta-glucosidase GII precursor, M60402 and M60403) showed reduced expression, whereas two genes functioning in RNA silencing (DQ321488 and DQ321489) remained unchanged (Figure 4B).

## Discussion

The transcriptome of RDV infected rice plants was profiled in this study. A number of genes are differentially expressed in RDV infected rice. Changes of most of these genes are consistent with previous studies carried out using *N. benthamiana* or *Arabidopsis thaliana *[4,10,11,35-39]. Also, we find induction of a set of defense related genes including PR genes, WRKY transcription factors and several genes functioning in RNA silencing. This is consistent with reports of Shimizu *et al. *[9] and Satoh *et al. *[7] showing that increased expression of defense related genes may be a common response of rice infected with RDV [7,40]. However, our results indicate that RDV induced the expression of far more genes than it suppressed. This is in sharp contrast to the report of Shimizu *et al.*[40]. Multiple resons may be responsible for the inconsistency. But the most plausible one is that transcriptome change in response to RDV infection is host-specific. In the study of Shimizu *et al. *[40], the *Japonica* subspecies of rice, namely *Oryza sativa L. cv. Nipponbare*, was used, whereas in this study, the *indica* subspecies of rice, namely *Oryza sativa* L. ssp. *indica* cv Yixiang2292, was used. Yixiang 2292, the rice variety used in this study, shows moderate resistance to RDV infection. It can develop typical symptoms of RDV infection, but the symptoms are not as severe as those of more susceptible varieties. A number of recent studies have demonstrated that there is a co-relation between symptom severity and transcriptional alteration in different virus-host combinations [6-8,41].

Many genes related to protein synthesis (Figure 1) were found and the GO term Ribosome was significantly enriched in the DEGs (Table 1). This is consistent with several studies showing that up-regulation of ribosomal genes and a set of other genes involved in protein synthesis could be a general response of plants to many viruses [10-12]. It has been suggested that this may be a strategy used by the virus to enhance the capacity of the cell to synthesize proteins [10-12].

As a two-subunit ribonucleoprotein complex comprising tens of ribosomal proteins and four species of ribosomal RNAs, the biogenesis of ribosome is one of the most energy consuming cellular processes [42-44]. So it is anticipated that synthesis of ribosomal components should be downregulated in response to environmental cues, as it has been shown in yeast and in Arabidopsis [45,46]. Therefore, increased expression of ribosomal genes in virus infected plants may be a result of specific virus-host interaction.

Here, we show that RDV infection also causes a significant alteration of many nucleolar genes (Table 1). The fact that RDV Pns11 has a nuclear localization signal and induces the expression of nucleolus-related genes in tobacco (Figure 4) and the observation that nucleoli seems to be affected in RDV infected rice (Figure 3) support the notion that this alteration is specific and may be useful for RDV. Nucleolus is the site of ribosomal RNA synthesis and processing and ribosome maturation [47]. Therefore, it is possible that, besides ribosome, RDV may also target nucleolus to manipulate the translation machinery of rice. Interestingly, there is evidence that certain nucleolar components or its overall state play a crucial role in controlling ribosomal gene expression and biogenesis [46,48]. So it is intriguing to speculate that RDV may specifically target nucleolus to enhance expression of ribosomal genes. In this regard, it is worth noting that a number of viruses, including many RNA viruses whose primary site of replication is the cytoplasm, encode special proteins to target nucleolus [49]. It would be very interesting to test the link between nucleolar targeting of these viruses and ribosome biogenesis of their hosts.

Besides ribosomal genes, malfunction of nucleolus may be responsible for altered expression of many other genes detected in this study. For example, emerging evidence suggests that nucleolus might play a role in the small interfering RNA (siRNA) pathway [47,50]. Therefore, many genes controlled by siRNAs may be altered because of malfunction of nucleolus in RDV infected rice. Consistent with this, we found a large number of genes encoding transposon/retrotransposon-related proteins in the DEGs (Figure 1). It is well known that tansposon or transposon-related genes are transcriptionally controlled by epigenetic modifications, in which siRNAs play an important role [51]. To our knowledge, altered expression of transposon/retrotransposon-related genes has never been reported in virus infected plants. However, we do not favor the possibility that this is specific to RDV. Instead, DEGs were classified automatically using web-based tools in most previous studies. In this way, transposon/retrotransposon-related genes tend to be classified into “unknown” genes and be excluded for further analysis.

## Materials and methods

### Sources of virus and insects

RDV Fujian isolate, China, was maintained in “Taizhong-1” rice plants grown in greenhouses at 25 ± 3°C, 55 ± 5% RH and under natural sunlight. Insects (*Nephotettix cincticeps*) source: high infectious green rice leafhoppers cultured in our lab with five generations of artificial rearing on rice seedlings.

### Plant growth and inoculation

Seeds (*Oryza sativa* L. ssp. *indica* cv Yixiang2292) were sowed and germinated on a pot (60 mm in diameter and 50 mm in height) that had been filled with commercial soil mixture (FAFARD SOILS, Southern Agricultural Insecticides Inc Palmetto, FL, 34221). Rice seedlings were subjected to a two-day inoculation using high infectious green rice leafhoppers or virus-free insects (for mock inoculation) by the one test tube-one-seedling method. Inoculated seedlings were transplanted to an iron dish filled with cultivation layer soil of experimental farmland. They were kept in a south-facing greenhouse at 25 ± 3°C with 55 ± 5% RH and under natural sunlight. The aerial parts of 8 entire rice plants were sampled randomly and pooled at 22 dpi, i.e., 7d after appearance of the symptom (the earliest symptoms, i.e. white chlorotic specks in newly developed leaves, appeared at approximately 15 dpi). The samples were flash-frozen in liquid nitrogen, and stored at -80°C for until use.

### RNA preparation and microarray hybridization and scanning

Total RNA was extracted from the virus- or mock-inoculated leaves with TRIzol reagent (Invitrogen). RNA was further purified using RNeasy columns (Qiagen, Valencia, CA, USA). An aliquot of 2 μg of total RNA was used to synthesize double-stranded cDNA, then produced biotin-tagged cRNA using MessageAmp™ II aRNA Amplification Kit. The resulting bio-tagged cRNA were fragmented to strands of 35 to 200 bases in length according to Affymetrix's protocols. The fragmented cRNA was hybridized to Affymetrix Rice Genome Array containing 51,279 transcripts which includes approximately 48,564 *japonica* transcripts and 1,260 transcripts representing the *indica* cultivar (http://www.affymetrix.com). Hybridization was performed at 45°C with rotation for 16 h (Affymetrix GeneChip Hybridization Oven 640). The GeneChip arrays were washed and then stained (streptavidin-phycoerythrin) on an Affymetrix Fluidics Station 450 followed by scanning on GeneChip Scanner 3000. We altogether used 6 chips to perform the analysis of 6 RNA samples.

### Microarray data analysis

Hybridization data were analyzed using GeneChip Operating software (GCOS 1.4). The scanned images were firstly assessed by visual inspection then analyzed to generate raw data files saved as CEL files using the default setting of GCOS 1.4. A global scaling procedure was performed to normalize the arrays using dChip software. In a comparison analysis, two class unpaired method was applied in the Significant Analysis of Microarray (SAM) software to identify significantly differentially expressed genes between Test group and Control group. All differentially expressed genes were analyzed using the web-based Molecular Annotation System 3.0 (MAS 3.0, http://bioinfo.capitalbio.com/mas/). MAS 2.0 integrate three different open source pathway resources-KEGG, BioCarta and GenMAPP. In the MAS 3.0 tool, the pathways and GO were ranked with statistical significance by calculating their *P*-values based on hypergeometric distribution. The GeneChip hybridization and scanning were performed at the Microarray Resource Laboratory at Beijing CapitalBio Corporation, Beijing, China, in which GeneChip microarray service was certificated by Affymetrix.

### Transient expression in leaves of N. benthamiana

Agro-infiltration for transient expression in leaves of *Nicotiana benthamiana*, Leuzinger was carried out as described [52]. Briefly, individual *Agrobacterium* GV3101 strains with different expression constructs (or empty vector as control) were co-infiltrated into *N. benthamiana* leaves using a syringe without needle. After 3 day of transient expression, leaves were harvested for RNA extraction.

### Real-time PCR assay

Total RNA used for verification of microarray data was prepared from plants that had been grown independently of those used for isolation of RNA for microarray analysis. One Step RNA PCR Kit (AMV) (TaKaRa, Japan) was used. Gene-specific primers were designed by Primer 5 (for a list of the primers used in this study, see Additional file 3: Table S3) and synthesized by Boya Company (Shanghai, China). Relative quantitation method was used. Rice UBQ11 gene and tobacco EF-1α were used as the control to normalize all data [53] (for a list of these genes primer sequence, see Additional file 3: Table S3).

### Electron microscopy

For electron microscopy experiments, RDV infected and health rice samples were fixed with 2.5% glutaraldehyde at 4°C overnight, washed in 0.1 M phosphate buffer (pH 7.0) for 3 times (15 min per time), and post-fixed in phosphate-buffered 1.0% OsO4 for 2 h. Then the tissues were buffer-washed, dehydrated with ethanol (50%, 70%, 80%, 90%, 95% and 100%) and embedded in Epon-Araldite. Ultrathin sections (70–90 nm) were cut with a Reichert ultra-microtome, stained with aqueous uranyl acetate and lead citrate, and examined with a Jeol JEM-1230 transmission electron microscope (Jeol, Tokyo, Japan).

## Competing interests

The authors declare that they have no competing interests.

## Authors’ contributions

Conceived and designed the experiments: JGW, ZJW and LHX. Performed the experiments and analyzed the data: JGW, LY, ZGD and KCW. Wrote the paper: JGW, ZGD, LY. All authors read and approved the final manuscript.

## Supplementary Material

Additional file 1: Table S1 RDV responsive genes. Click here for file

Additional file 2: Table S2 Functional classifications of RDV responsive genes. Click here for file

Additional file 3: Table S3 The primers used in this study. Click here for file
