# Supplementary material for: Transcriptome profiling confirmed correlations between symptoms and transcriptional changes in RDV infected rice and revealed nucleolus as a possible target of RDV manipulation
Source: Virol J. 2014 May 6;11:81. doi: 10.1186/1743-422X-11-81 (PMC4032362; doi:10.1186/1743-422X-11-81)
Supplement: Additional file 2: Table S2 — Functional classifications of RDV responsive genes. [file 1743-422X-11-81-S2.docx]

| **Supplemental Table S2. Functional classifications of RDV responsive genes** | | | | |
| --- | --- | --- | --- | --- |
|  |  |  |  |  |
| **Probe Set ID** | **Representative Public ID** | **Fold Change** | **q-value(%)** | **Description** |
|  |  |  |  |  |
| **Stress/defense related genes** | | | | |
|  |  |  |  |  |
| Os.33131.1.A1_at | AK101653.1 | 2.21163 | 0 | WRKY DNA binding domain containing protein, expressed |
| Os.54467.1.S1_at | AK105244.1 | 2.57672 | 0 | WRKY DNA binding domain containing protein |
| Os.4844.1.S1_at | AK067416.1 | 6.09419 | 0 | Peroxidase family protein, expressed |
| Os.6291.1.S1_x_at | AK108191.1 | 6.03799 | 0 | Pathogenesis-related protein 5 precursor, putative, expressed |
| Os.19861.1.S1_at | AF306651.1 | 9.33045 | 0 | Pathogenesis-related protein 1 precursor, putative, expressed |
| OsAffx.30135.1.S1_at | 9637.m02946 | 3.03297 | 0 | NB-ARC domain containing protein, expressed |
| Os.8586.1.S1_at | AK103914.1 | 4.84954 | 0 | Leucine-rich repeat transmembrane protein kinase, putative, expressed |
| Os.54530.1.S1_x_at | AK105531.1 | 7.10471 | 0 | Leucine-rich repeat family protein, putative, expressed |
| OsAffx.17422.1.S1_at | 9636.m04000 | 5.74716 | 0 | Leucine Rich Repeat family protein, expressed |
| Os.7637.1.S1_at | AK102922.1 | 6.21453 | 0 | L-ascorbate oxidase homolog precursor, putative, expressed |
| Os.22957.1.S1_at | AK064612.1 | 2.54703 | 0 | Glutathione S-transferase, N-terminal domain containing protein, expressed |
| Os.40000.1.S1_at | CR279062 | 4.52645 | 0 | Glutathione S-transferase GSTU6, putative, expressed |
| Os.27963.1.A1_at | AK063053.1 | 2.71925 | 0 | ABA/WDS induced protein, expressed |
| Os.6116.1.S1_x_at | CF329533 | 2.0963 | 0 | Thaumatin-like cytokinin-binding protein, putative, expressed |
| Os.46809.1.S1_at | AK110572.1 | 2.49826 | 0 | Thaumatin family protein, expressed |
| Os.10651.1.S1_at | AK121915.1 | 3.62012 | 0 | Gamma-thionins family protein, expressed |
| Os.20289.1.S1_at | AY435041.1 | 4.19394 | 0 | Barwin, putative, expressed |
| Os.15355.1.S1_x_at | AK067000.1 | 0.36386 | 0.4278058 | Cationic peroxidase 1 precursor, putative, expressed |
| Os.54530.1.S1_at | AK105531.1 | 4.26171 | 0.4278058 | Leucine-rich repeat family protein, putative, expressed |
| OsAffx.24011.1.S1_at | 9629.m07137 | 1.69724 | 0.4278058 | Glutathione S-transferase, putative, expressed |
| Os.6116.1.S1_at | CF329533 | 2.05549 | 0.4278058 | thaumatin-like cytokinin-binding protein, putative, expressed |
| Os.7372.1.S1_at | AU100845 | 1.89715 | 0.8078121 | ABA/WDS induced protein, expressed |
| Os.50838.1.A1_at | AK058886.1 | 4.24803 | 1.1122951 | Peroxidase 45 precursor, putative, expressed |
| Os.11547.1.S1_s_at | AK064918.1 | 2.27343 | 1.1122951 | Peroxidase 16 precursor, putative, expressed |
| Os.6291.1.S1_at | AK108191.1 | 1.96944 | 1.1122951 | Pathogenesis-related protein 5 precursor, putative, expressed |
| Os.46475.1.S1_at | AK073088.1 | 2.49713 | 1.1122951 | Leucine Rich Repeat family protein, expressed |
| Os.23119.1.S1_at | AK059226.1 | 3.10665 | 1.1122951 | Glutathione S-transferase, C-terminal domain containing protein, expressed |
| Os.8178.1.S1_at | AY050642.1 | 4.49525 | 1.1122951 | Win1 precursor, putative, expressed |
| Os.27488.1.S1_at | AK120815.1 | 2.15317 | 1.5448543 | Leucine-rich repeat transmembrane protein kinase, putative, expressed |
| Os.49496.1.S1_at | AK071465.1 | 1.71193 | 1.5448543 | L-ascorbate oxidase homolog precursor, putative, expressed |
| Os.40417.1.A1_at | CF337705 | 2.05649 | 1.5448543 | Harpin-induced protein 1 containing protein, expressed |
| Os.53852.1.S1_at | AK100406.1 | 1.91122 | 1.5448543 | Glutathione-conjugate transporter, putative, expressed |
| Os.1406.1.S1_at | AK060687.1 | 3.51003 | 1.8606407 | Peroxidase family protein, expressed |
| Os.38022.1.S1_at | AK064650.1 | 2.25567 | 1.8606407 | Glutathione S-transferase, N-terminal domain containing protein, expressed |
| Os.14372.1.S1_at | AK102889.1 | 3.20306 | 1.8606407 | Glutathione S-transferase, N-terminal domain containing protein, expressed |
| Os.55597.1.S1_at | AK108389.1 | 3.11813 | 2.3550221 | WRKY DNA binding domain containing protein, expressed |
|  |  |  |  |  |
| **Probe Set ID** | **Representative Public ID** | **Fold Change** | **q-value(%)** | **Description** |
| Os.13965.1.S1_at | AK121108.1 | 3.63432 | 2.3550221 | Pathogenesis-related protein PRMS precursor, putative, expressed |
| Os.55089.1.S1_at | AK107463.1 | 1.98881 | 2.3550221 | Harpin-induced protein 1 containing protein, expressed |
| OsAffx.15035.1.S1_at | 9633.m03737 | 2.0753 | 2.4099727 | WRKY DNA binding domain containing protein |
| Os.32680.1.S1_at | AK103262.1 | 6.73134 | 2.6108037 | Leucine-rich repeat family protein, putative, expressed |
| Os.50552.1.S1_at | AK121795.1 | 1.82225 | 2.6108037 | Leucine Rich Repeat family protein, expressed |
| Os.31431.1.S1_at | AK107346.1 | 2.26136 | 2.6108037 | Hypersensitive-induced response protein, putative, expressed |
| Os.21895.1.S1_at | CA758110 | 3.32024 | 2.6108037 | Early salt stress and cold acclimation-induced protein 2-1, putative, expressed |
| Os.9445.1.S1_at | C99054 | 2.39437 | 3.0257366 | Wound induced protein, putative, expressed |
| Os.159.1.S1_s_at | D14482.1 | 2.49361 | 3.0257366 | Peroxidase N precursor, putative, expressed |
| Os.11558.1.S1_at | AK101508.1 | 2.01914 | 3.0257366 | Peroxidase family protein, expressed |
| OsAffx.26383.1.S1_at | 9632.m03698 | 1.51096 | 3.0257366 | Peroxidase 65 precursor, putative, expressed |
| OsAffx.28164.1.S1_at | 9634.m04685 | 2.52754 | 3.0257366 | Peroxidase 16 precursor, putative, expressed |
| Os.11563.1.S1_at | AK108713.1 | 3.79962 | 3.0257366 | Peroxidase 1 precursor, putative, expressed |
| Os.53228.1.S1_at | AK071464.1 | 1.85019 | 3.0257366 | Salicylic acid-induced protein 19, putative, expressed |
| Os.12387.1.S1_at | AK119676.1 | 3.72367 | 3.0257366 | Pathogenesis-related protein Bet v I family protein, expressed |
| Os.32417.1.S1_at | AK105103.1 | 2.63381 | 3.0257366 | Leucine Rich Repeat family protein, expressed |
| Os.11945.1.S1_at | AK111606.1 | 1.63689 | 3.0765608 | WRKY DNA binding domain containing protein, expressed |
| Os.5299.2.S1_a_at | AK073600.1 | 1.54329 | 3.0765608 | Universal stress protein family protein, expressed |
| Os.12207.1.S1_at | AK102299.1 | 1.70624 | 3.101945 | leucine-rich repeat family protein, putative, expressed |
| Os.32679.1.S1_at | AK059532.1 | 1.64046 | 3.101945 | Leucine Rich Repeat family protein, expressed |
| Os.7832.1.S1_at | AK103660.1 | 1.83612 | 4.3419899 | Peroxidase family protein, expressed |
| Os.11548.1.S1_at | AK071181.1 | 2.12467 | 4.3419899 | Peroxidase 35 precursor, putative, expressed |
| Os.15982.1.S1_s_at | BI800939 | 1.61179 | 4.3419899 | NB-ARC domain containing protein, expressed |
| Os.49215.1.S2_at | AK105421.1 | 2.26672 | 4.3419899 | Leucine Rich Repeat family protein, expressed |
| Os.9101.1.S1_at | AF402797.1 | 1.58809 | 4.3419899 | Glutathione S-transferase GSTU6, putative, expressed |
| Os.52605.1.S1_at | AK068276.1 | 2.2959 | 4.3419899 | Thaumatin family protein, expressed |
| Os.53486.1.S1_at | AK072906.1 | 1.90038 | 4.8372211 | WRKY family transcription factor, putative, expressed |
| Os.13011.1.S1_at | AB059567.1 | 1.863 | 4.8372211 | Leucine-rich repeat protein kinase, putative |
| Os.45423.1.S1_at | NM_184266.1 | 2.13385 | 4.8372211 | Leucine Rich Repeat family protein, expressed |
| Os.4294.1.S1_a_at | AY332474.1 | 2.198 | 4.8372211 | Leucine Rich Repeat family protein, expressed |
| OsAffx.14585.1.S1_s_at | 9633.m00699 | 2.67099 | 4.8372211 | Leucine Rich Repeat family protein, expressed |
| Os.15269.1.S1_at | AK059955.1 | 1.85734 | 4.8372211 | Glutathione S-transferase, C-terminal domain containing protein, expressed |
| Os.40000.1.S1_x_at | CR279062 | 1.68501 | 4.8372211 | Glutathione S-transferase GSTU6, putative, expressed |
| Os.55402.1.S1_at | AK108017.1 | 2.41528 | 4.8372211 | Glutaredoxin-like family protein, expressed |
| Os.1153.1.S1_at | AK102117.1 | 2.11542 | 5.8786028 | Peroxidase 72 precursor, putative, expressed |
| OsAffx.19161.1.S1_at | 9639.m03133 | 1.90582 | 5.8786028 | NB-ARC domain containing protein, expressed |
| Os.47706.2.A1_at | CB655576 | 1.67252 | 5.8786028 | NB-ARC domain containing protein |
| Os.45953.1.S1_at | NM_192974.1 | 2.24268 | 5.8786028 | Leucine Rich Repeat family protein, expressed |
| Os.53377.1.S1_at | AK072348.1 | 2.55023 | 5.8786028 | Leucine Rich Repeat family protein, expressed |
| Os.2998.1.S1_at | AK102587.1 | 1.56135 | 5.8786028 | Early-responsive to dehydration protein, putative, expressed |
| **Probe Set ID** | **Representative Public ID** | **Fold Change** | **q-value(%)** | **Description** |
| Os.2225.1.S1_at | AF090698.1 | 1.87915 | 5.8786028 | Elicitor-responsive protein 3, putative, expressed |
| Os.25329.1.A1_at | AK069182.1 | 1.98474 | 5.8786028 | Thaumatin-like protein precursor, putative, expressed |
| Os.11997.1.S1_at | AK105051.1 | 1.77091 | 5.8786028 | Thioredoxin-like 1, putative, expressed |
| **Signal transduction** | | | | |
|  |  |  |  |  |
| Os.28514.1.S1_at | AK111442.1 | 4.59927 | 0 | Calmodulin-binding protein, putative, expressed |
| OsAffx.5948.1.S1_at | 9636.m02685 | 4.20554 | 0 | Calmodulin-binding protein, putative |
| Os.27808.1.S1_s_at | AK067697.1 | 1.72105 | 0 | Calmodulin, putative, expressed |
| Os.26870.1.S1_at | AK066121.1 | 0.49893 | 0.4278058 | Receptor-like protein kinase 2, putative, expressed |
| Os.50881.1.S1_x_at | AK059199.1 | 2.61241 | 0.4278058 | Protein kinase domain containing protein, expressed |
| Os.27110.1.A1_at | AK105217.1 | 3.10534 | 0.4278058 | Protein kinase domain containing protein, expressed |
| Os.11301.1.S1_x_at | AK070778.1 | 2.01203 | 0.4278058 | EF hand family protein, expressed |
| Os.11301.1.S1_at | AK070778.1 | 2.52179 | 0.4278058 | EF hand family protein, expressed |
| Os.455.1.S1_at | CF321434 | 1.6342 | 0.4278058 | Calmodulin, putative, expressed |
| Os.27808.1.S1_at | AK067697.1 | 1.70408 | 1.1122951 | Calmodulin, putative, expressed |
| OsAffx.12547.1.S1_at | 9630.m04699 | 2.64773 | 1.5448543 | Protein kinase domain containing protein, expressed |
| Os.54825.1.S1_at | AK106842.1 | 6.12398 | 1.5448543 | Protein kinase domain containing protein, expressed |
| Os.27767.1.A1_s_at | CB632726 | 2.15809 | 1.5448543 | Calcium binding EGF domain containing protein, expressed |
| Os.49654.1.S1_at | AK101878.1 | 2.39941 | 1.8606407 | Protein phosphatase 2C, putative, expressed |
| Os.10384.1.S1_at | AK072292.1 | 1.91945 | 1.8606407 | protein phosphatase 2C family protein, putative, expressed |
| Os.46804.2.S1_at | NM_197767.1 | 1.99472 | 1.8606407 | Protein kinase domain containing protein, expressed |
| Os.35858.2.S1_at | NM_192941.1 | 2.36116 | 1.8606407 | Protein kinase domain containing protein, expressed |
| Os.9764.1.S1_at | AK110372.1 | 2.13284 | 1.8606407 | Calmodulin-binding protein, putative, expressed |
| Os.2000.2.S1_x_at | AU030308 | 1.98217 | 2.3550221 | protein kinase family protein, putative, expressed |
| Os.26382.1.S1_at | CB680782 | 1.51076 | 2.3550221 | Protein kinase domain containing protein, expressed |
| OsAffx.12863.1.S1_s_at | 9631.m01412 | 1.66319 | 2.4099727 | Protein kinase domain containing protein, expressed |
| Os.27797.1.A1_at | CB658382 | 3.80594 | 2.6108037 | IQ calmodulin-binding motif family protein, expressed |
| Os.27092.1.S1_at | AK067073.1 | 1.88421 | 3.0257366 | Protein kinase domain containing protein, expressed |
| Os.35858.2.S1_x_at | NM_192941.1 | 2.08301 | 3.0257366 | Protein kinase domain containing protein, expressed |
| Os.35858.1.S1_at | AK065494.1 | 2.50243 | 3.0257366 | Protein kinase domain containing protein, expressed |
| Os.55572.1.S1_at | AK108339.1 | 2.91218 | 3.0257366 | Protein kinase domain containing protein, expressed |
| Os.26870.2.A1_x_at | CB623213 | 0.41652 | 3.0765608 | Receptor-like protein kinase 2, putative, expressed |
| Os.49690.1.S1_s_at | AF193835.1 | 1.9246 | 3.0765608 | Receptor protein kinase CLAVATA1 precursor, putative, expressed |
| Os.6595.2.S1_s_at | AK060612.1 | 2.10283 | 3.0765608 | Protein kinase family protein, putative, expressed |
| Os.54119.1.S1_at | AK102046.1 | 2.31677 | 3.0765608 | Protein kinase family protein, putative, expressed |
| Os.8102.1.S1_at | AK103438.1 | 2.16089 | 3.0765608 | IQ calmodulin-binding motif family protein, expressed |
| Os.23124.1.S1_at | AK062764.1 | 2.04077 | 3.0765608 | Calmodulin-binding protein, putative, expressed |
| Os.46832.1.S1_at | AK109037.1 | 2.01396 | 3.0765608 | Calcium-transporting ATPase 13, plasma membrane-type, putative, expressed |
| Os.54625.1.S1_at | AK106290.1 | 2.70598 | 3.101945 | Serine/threonine protein kinase, putative, expressed |
| Os.26888.1.A1_s_at | CB626658 | 1.69689 | 3.101945 | Protein kinase domain containing protein, expressed |
| Os.49855.1.S1_at | AK112048.1 | 1.54082 | 3.101945 | Protein kinase domain containing protein, expressed |
| **Probe Set ID** | **Representative Public ID** | **Fold Change** | **q-value(%)** | **Description** |
| Os.15688.1.S1_at | AK067865.1 | 1.54927 | 3.101945 | Calmodulin-binding protein, putative, expressed |
| Os.18316.1.S1_at | AK073557.1 | 1.96262 | 4.3419899 | Protein kinase family protein, putative, expressed |
| Os.49805.1.S1_at | AK111655.1 | 1.59244 | 4.3419899 | Protein kinase domain containing protein, expressed |
| Os.24008.1.S1_at | AK105254.1 | 1.61382 | 4.3419899 | Protein kinase domain containing protein, expressed |
| Os.54508.1.S1_s_at | AK105486.1 | 1.66399 | 4.3419899 | IQ calmodulin-binding motif family protein, expressed |
| Os.49452.1.S1_at | AK070336.1 | 2.0037 | 4.3419899 | Glycerophosphoryl diester phosphodiesterase 1 precursor, putative, expressed |
| Os.9073.1.S1_at | AK109278.1 | 1.8954 | 4.3419899 | EF hand family protein, expressed |
| Os.54251.1.S1_at | AK102803.1 | 1.63865 | 4.3419899 | NPH3 family protein, expressed |
| Os.22907.1.S1_at | AK107217.1 | 1.73854 | 5.8786028 | Protein kinase, putative, expressed |
| Os.32994.1.S1_at | AK102156.1 | 1.67313 | 5.8786028 | Protein kinase family protein, putative, expressed |
| Os.17036.1.S1_x_at | AK106429.1 | 1.90944 | 5.8786028 | Protein kinase family protein, putative, expressed |
| Os.4149.2.S1_x_at | AK062249.1 | 1.62715 | 5.8786028 | Protein kinase domain containing protein, expressed |
| Os.53070.1.S1_at | AK070694.1 | 1.63119 | 5.8786028 | Protein kinase domain containing protein, expressed |
| OsAffx.16125.1.S1_at | 9635.m00597 | 1.65409 | 5.8786028 | Protein kinase domain containing protein |
| Os.9721.1.S1_at | AK067883.1 | 1.78511 | 5.8786028 | MAP3K-like protein kinase, putative, expressed |
| Os.27581.1.A1_at | CB678589 | 1.66904 | 5.8786028 | IQ calmodulin-binding motif family protein, expressed |
| Os.27474.1.S1_at | AK066537.1 | 1.97971 | 5.8786028 | SHR5-receptor-like kinase, putative, expressed |
| **Transporter/channel** | | | | |
|  |  |  |  |  |
| Os.15532.1.S1_at | AK121690.1 | 1.85549 | 1.1122951 | Multidrug resistance associated protein MRP2, putative, expressed |
| Os.52796.2.S1_x_at | AK069311.1 | 3.30624 | 1.5448543 | Subtilisin N-terminal Region family protein, expressed ,Ammonium transporter 2, putative, expressed |
| Os.46480.1.S1_at | AK072452.1 | 2.71358 | 1.5448543 | ABC-2 type transporter family protein, expressed |
| Os.15732.1.S1_s_at | AU032700 | 1.56293 | 2.6108037 | Zinc transporter, putative, expressed |
| Os.27207.1.S1_at | AK101523.1 | 2.04063 | 3.0257366 | Chloride channel protein CLC-a, putative, expressed |
| Os.27080.1.S1_at | AK068280.1 | 1.62104 | 4.8372211 | Sugar transporter family protein, expressed |
| Os.5349.1.S1_at | AK121517.1 | 1.80731 | 4.8372211 | ABC transporter, putative, expressed |
| Os.47982.1.A1_x_at | AK058355.1 | 2.3196 | 5.8786028 | ABC transporter, putative, expressed |
| **Metabolism** | | | | |
|  |  |  |  |  |
| Os.28124.1.S1_at | AK059767.1 | 2.58711 | 0 | Glycosyl hydrolases family 18 protein, expressed |
| Os.46472.1.S1_at | AK073394.1 | 2.00064 | 0 | Glycosyl hydrolases family 17 protein, expressed |
| Os.7086.1.S1_at | BI305420 | 5.52812 | 0 | Glycosyl hydrolases family 17 protein, expressed |
| Os.7566.1.S1_at | AK059277.1 | 9.77848 | 0 | Glycosyl hydrolases family 16 protein, expressed |
| Os.21839.1.S1_at | AK070299.1 | 1.50264 | 0 | 2-Hydroxyisoflavanone dehydratase, putative, expressed |
| OsAffx.16247.1.S1_at | 9635.m01363 | 1.65161 | 0 | UDP-glucoronosyl and UDP-glucosyl transferase family protein, expressed |
| Os.10597.1.S1_at | AK102415.1 | 4.67352 | 0 | UDP-glucoronosyl and UDP-glucosyl transferase family protein, expressed |
| OsAffx.32262.1.A1_at | X15901.1 | 0.52363 | 0.4278058 | Nicotinate-nucleotide pyrophosphorylase family protein, expressed |
| OsAffx.13994.1.S1_at | 9632.m02584 | 17.9719 | 0.8078121 | Terpene synthase family, metal binding domain containing protein |
| Os.9427.1.S1_at | AK067458.1 | 2.01528 | 0.8078121 | Cytochrome P450 family protein, expressed |
| Os.28952.1.S1_at | AK110852.1 | 2.2121 | 0.8078121 | Cytochrome P450 family protein, expressed |
| **Probe Set ID** | **Representative Public ID** | **Fold Change** | **q-value(%)** | **Description** |
| Os.23411.1.S1_at | AK101968.1 | 3.82861 | 1.1122951 | Fatty acid desaturase family protein, expressed |
| Os.51053.1.S1_at | AK102820.1 | 3.7999 | 1.5448543 | Glycosyl hydrolases family 18 protein, expressed |
| Os.10200.1.S1_at | CB620205 | 1.55641 | 1.5448543 | Cytochrome P450 89A2, putative, expressed |
| Os.17014.1.S1_s_at | AK067411.1 | 1.60617 | 1.8606407 | NAD dependent epimerase/dehydratase family protein, expressed |
| Os.27794.1.S1_at | AK071447.1 | 4.98719 | 2.3550221 | Terpene synthase family, metal binding domain containing protein, expressed |
| Os.17111.2.S1_x_at | AK061650.1 | 1.73531 | 2.3550221 | Cytochrome P450 family protein, expressed |
| Os.10360.1.S1_at | AK099925.1 | 1.73693 | 2.3550221 | Cytochrome P450 family protein, expressed |
| Os.23185.1.S1_at | NM_195030.1 | 2.03376 | 2.3550221 | Cytochrome P450 family protein, expressed |
| Os.23542.1.A1_at | AK100115.1 | 1.64005 | 2.6108037 | Glyoxysomal fatty acid beta-oxidation multifunctional protein MFP-a, putative, expressed |
| Os.17181.1.S1_at | AK105785.1 | 2.0863 | 2.6108037 | UDP-glucoronosyl and UDP-glucosyl transferase family protein, expressed |
| OsAffx.21499.1.S1_at | NM_192066.1 | 1.54848 | 3.0257366 | Phosphatidyl serine synthase family protein |
| Os.49566.1.S1_at | AK110659.1 | 1.6615 | 3.0257366 | Cytochrome P450 89A2, putative, expressed |
| Os.7150.1.S1_at | AK101104.1 | 1.68099 | 3.0257366 | 3-hydroxy-3-methylglutaryl-coenzyme A reductase 3, putative, expressed |
| OsAffx.32263.1.A1_at | X15901.1 | 0.49732 | 3.0765608 | Glyoxalase family protein, expressed , nicotinate-nucleotide pyrophosphorylase family protein, expressed |
| Os.26716.1.S1_at | AK102449.1 | 5.38029 | 3.0765608 | Glycosyl hydrolase family 20, catalytic domain containing protein, expressed |
| Os.32736.1.S1_at | AK103085.1 | 2.2581 | 3.0765608 | Fatty acid hydroxylase family protein, expressed |
| Os.5844.1.S1_at | AK100641.1 | 2.01981 | 3.101945 | Glycosyltransferase QUASIMODO1, putative, expressed |
| Os.16235.1.S1_at | AK108140.1 | 1.53567 | 3.101945 | Glycosyltransferase 6, putative, expressed |
| Os.15914.1.S1_at | AK105382.1 | 1.82936 | 3.101945 | Glycosyl transferase family 20 protein, putative, expressed |
| Os.10574.1.S1_at | AU029726 | 1.83833 | 3.101945 | Glycosyl hydrolases family 16 protein, expressed |
| Os.38849.1.S1_at | AK119887.1 | 2.01513 | 3.101945 | Glycosyl hydrolase family 3 C terminal domain containing protein, expressed |
| Os.13708.2.A1_at | CB655990 | 2.19974 | 3.101945 | Glycosyl hydrolase family 14 protein, expressed |
| Os.25621.2.S1_at | AK071599.1 | 1.78429 | 3.101945 | Cytochrome P450 family protein, expressed |
| Os.51066.1.S1_at | AK060486.1 | 2.22114 | 3.101945 | Cytochrome P450 family protein, expressed |
| Os.8021.1.S1_x_at | AK071033.1 | 3.32499 | 3.101945 | Cytochrome P450 86A2, putative, expressed |
| Os.10348.1.S1_at | AK063325.1 | 1.57029 | 3.101945 | UDP-glucoronosyl and UDP-glucosyl transferase family protein, expressed |
| Os.23472.2.A1_x_at | NM_190222.1 | 3.17076 | 4.3419899 | Glycosyl hydrolase family 20, catalytic domain containing protein, expressed |
| Os.32141.1.S1_at | AK120790.1 | 2.33721 | 4.3419899 | Glycosyl hydrolase family 1 protein, expressed |
| Os.20572.2.S1_at | AK065109.1 | 2.6795 | 4.3419899 | Terpene synthase family, metal binding domain containing protein, expressed |
| Os.55539.1.S1_at | AK108283.1 | 1.92321 | 4.3419899 | NAD dependent epimerase/dehydratase family protein, expressed |
| OsAffx.2362.1.S1_s_at | 9629.m05792 | 1.65453 | 4.3419899 | Cytochrome P450 family protein, expressed |
| Os.770.1.S1_at | AK101667.1 | 1.65231 | 4.3419899 | Cytochrome P450 72A1, putative, expressed |
| OsAffx.29875.1.S1_s_at | 9637.m01388 | 1.64177 | 4.3419899 | Cytochrome b5-like Heme/Steroid binding domain containing protein, expressed |
| Os.12579.1.S1_at | AK059654.1 | 1.54391 | 4.3419899 | Acetyl-CoA acetyltransferase, cytosolic 1, putative, expressed |
| Os.26862.1.S1_s_at | CB650128 | 0.64118 | 4.3419899 | Saccharopine dehydrogenase family protein, expressed |
| Os.14153.2.S1_x_at | AK109443.2 | 3.51081 | 4.8372211 | Transferase family protein, expressed |
| Os.17012.1.S1_at | BI811979 | 1.51399 | 4.8372211 | Acyltransferase family protein |
| **Probe Set ID** | **Representative Public ID** | **Fold Change** | **q-value(%)** | **Description** |
| Os.51918.1.S1_at | AK064276.1 | 1.83947 | 4.8372211 | Copine family protein, expressed , Glucan endo-1,3-beta-glucosidase precursor, putative, expressed |
| Os.49475.1.S1_at | AK120709.1 | 2.52247 | 5.8786028 | Glycosyl hydrolases family 17 protein, expressed |
| Os.14153.1.S1_at | AK063515.1 | 2.59042 | 5.8786028 | Transferase family protein, expressed |
| Os.46457.1.S1_at | AK100207.1 | 4.68188 | 5.8786028 | Transferase family protein, expressed |
| Os.53946.1.S1_at | AK100965.1 | 1.71922 | 5.8786028 | NAD dependent epimerase/dehydratase family protein, expressed |
| Os.16786.1.S1_at | AK068907.1 | 1.66004 | 5.8786028 | Cytochrome P450 family protein, expressed |
| **Protein fate** | | | | |
|  |  |  |  |  |
| OsAffx.29477.1.S1_x_at | 9636.m03258 | 2.48655 | 0 | U-box domain containing protein, expressed |
| Os.52965.1.S1_at | AK070159.1 | 2.78076 | 0 | Eukaryotic aspartyl protease family protein, expressed |
| Os.7771.1.S1_at | AK103715.1 | 9.19773 | 0 | Protease inhibitor/seed storage/LTP family protein, expressed |
| Os.41335.2.S1_x_at | NM_192679.1 | 2.92209 | 0 | ATPase, AAA family protein, expressed |
| OsAffx.28169.1.S1_at | 9634.m04712 | 6.43158 | 0 | ATPase, AAA family protein, expressed |
| Os.2081.1.S1_at | AK109883.1 | 10.1447 | 0 | ATPase, AAA family protein, expressed |
| Os.54109.1.S1_at | AK101987.1 | 22.7722 | 0 | ATPase, AAA family protein, expressed |
| Os.5242.1.S1_at | AF200467.1 | 7.05587 | 0 | Subtilisin N-terminal Region family protein, expressed |
| Os.6125.1.S1_at | AK069583.1 | 4.07143 | 0 | PDI, putative, expressed |
| OsAffx.30103.1.S1_s_at | 9631.m01483 | 3.68875 | 0.4278058 | Ubiquitin, putative, expressed |
| Os.30983.1.S1_at | AK108026.1 | 1.53528 | 0.4278058 | Zinc finger, C3HC4 type family protein, expressed |
| OsAffx.30103.4.S1_s_at | 9637.m02463 | 5.24556 | 1.1122951 | UBiQuitin family member, putative, expressed |
| Os.10403.1.S1_at | AK101646.1 | 3.93603 | 1.1122951 | Cucumisin-like serine protease, putative, expressed |
| OsAffx.29968.1.S1_s_at | 9637.m01880 | 1.80787 | 1.5448543 | U-box domain containing protein, expressed |
| Os.6150.1.S1_s_at | CF322516 | 1.90217 | 1.5448543 | PDI, putative, expressed |
| Os.49728.1.S1_at | AK102217.1 | 1.51482 | 1.8606407 | F-box domain containing protein, expressed , hypothetical protein |
| Os.18254.1.A1_at | AK062549.1 | 2.10016 | 1.8606407 | UBA/TS-N domain containing protein, expressed |
| Os.3046.1.S1_at | AK101853.1 | 1.9288 | 2.6108037 | Ubiquitin family protein, expressed |
| Os.5812.1.S1_at | AK071172.1 | 1.78733 | 2.6108037 | F-box domain containing protein, expressed |
| Os.46987.1.S1_x_at | CK738634 | 3.90018 | 2.6108037 | Eukaryotic aspartyl protease family protein, expressed |
| Os.13246.1.S1_at | AK061173.1 | 9.55899 | 3.0257366 | Protease inhibitor/seed storage/LTP family protein, expressed |
| Os.14208.1.S1_at | AK104980.1 | 2.76864 | 3.0257366 | Carboxyl-terminal proteinase, putative, expressed |
| Os.40947.1.A1_at | CB684734 | 2.00279 | 3.0765608 | Carboxyl-terminal proteinase, putative, expressed |
| Os.47906.1.A1_at | AK105838.1 | 4.01167 | 3.101945 | Protease inhibitor/seed storage/LTP family protein, expressed |
| Os.4693.1.S1_at | AK065137.1 | 1.77558 | 3.101945 | Kelch repeat-containing F-box family protein, putative, expressed |
| Os.6633.1.S1_at | AK103449.1 | 2.33468 | 3.101945 | Carboxyl-terminal peptidase, putative, expressed |
| Os.18743.1.S1_at | AK070376.1 | 1.92323 | 3.101945 | Subtilase family protein, putative, expressed |
| OsAffx.5699.1.S1_at | 9636.m00372 | 1.5311 | 4.3419899 | U-box domain containing protein, expressed |
| Os.6442.1.S1_at | AK062232.1 | 4.14926 | 4.3419899 | Eukaryotic aspartyl protease family protein, expressed |
| **Probe Set ID** | **Representative Public ID** | **Fold Change** | **q-value(%)** | **Description** |
| Os.49519.1.S1_at | AK063637.1 | 6.97964 | 4.3419899 | Eukaryotic aspartyl protease family protein, expressed |
| Os.7242.1.S1_at | AK073569.1 | 3.70645 | 4.3419899 | Protease inhibitor/seed storage/LTP family protein, expressed |
| Os.5492.1.S1_at | AK103109.1 | 1.61387 | 4.3419899 | Peptidyl-prolyl cis-trans isomerase, putative, expressed |
| Os.10872.1.S1_at | AK065505.1 | 2.33362 | 4.3419899 | ATPase, AAA family protein, expressed |
| Os.49157.1.S1_s_at | AK110974.1 | 3.23112 | 4.3419899 | Papain family cysteine protease containing protein, expressed |
| OsAffx.13431.1.S1_at | 9631.m04885 | 1.6024 | 4.8372211 | F-box domain containing protein, expressed |
| Os.46561.2.S1_at | AK063758.1 | 1.96673 | 4.8372211 | F-box domain containing protein, expressed |
| OsAffx.25751.1.S1_s_at | 9631.m05696 | 2.15726 | 4.8372211 | Protease inhibitor/seed storage/LTP family protein, expressed |
| Os.22596.1.S1_s_at | CF309035 | 2.74817 | 4.8372211 | Zinc finger, C3HC4 type family protein, expressed |
| Os.31545.1.S1_at | AK107155.1 | 1.75329 | 5.8786028 | Ubiquitin ligase SINAT5, putative, expressed |
| Os.38856.1.A1_s_at | CR290649 | 1.76252 | 5.8786028 | Ubiquitin family protein, putative, expressed |
| Os.2431.1.S1_at | AK106902.1 | 1.73374 | 5.8786028 | F-box domain containing protein, expressed |
| Os.14214.1.S1_at | AK066409.1 | 3.45181 | 5.8786028 | Eukaryotic aspartyl protease family protein, expressed |
| Os.9263.1.S1_at | AK070316.1 | 1.86684 | 5.8786028 | Prolyl oligopeptidase family protein, expressed |
| Os.24913.1.A1_at | AK071401.1 | 1.80833 | 5.8786028 | Zinc finger, C3HC4 type family protein, expressed |
| Os.212.1.S1_at | AK071739.1 | 1.94254 | 5.8786028 | Zinc finger, C3HC4 type family protein, expressed |
| Os.22596.1.S1_at | CF309035 | 2.97689 | 5.8786028 | Zinc finger, C3HC4 type family protein, expressed |
| Os.9533.1.S1_at | AK069220.1 | 1.65803 | 5.8786028 | Subtilisin N-terminal Region family protein, expressed |
| Os.22197.1.S1_at | AK099262.1 | 1.80061 | 5.8786028 | Serine hydrolase, putative, expressed |
| Os.28964.1.S1_at | AK110832.1 | 2.61605 | 5.8786028 | Papain family cysteine protease containing protein, expressed |
| **Cell wall function** | | | | |
|  |  |  |  |  |
| Os.8638.1.S1_at | BI805240 | 33.5684 | 0 | Proline-rich protein, putative, expressed |
| Os.8638.1.S1_s_at | BI805240 | 39.3857 | 0 | Proline-rich protein, putative, expressed |
| Os.10494.1.S1_at | AK059681.1 | 3.23691 | 0 | Glucan endo-1,3-beta-glucosidase precursor, putative, expressed |
| Os.1385.2.S1_x_at | NM_189714.1 | 2.71198 | 0 | Glucan endo-1,3-beta-glucosidase GII precursor, putative, expressed |
| Os.17479.1.S1_at | AK106178.1 | 2.19446 | 0 | Acidic endochitinase precursor, putative, expressed |
| Os.171.1.S1_at | AB026998.1 | 8.04254 | 0 | Acidic endochitinase precursor, putative, expressed |
| OsAffx.15187.1.S1_at | 9633.m04802 | 3.52583 | 0.4278058 | Polygalacturonase family protein, expressed |
| Os.1385.1.S1_at | AF443600.1 | 6.20097 | 0.4278058 | Glucan endo-1,3-beta-glucosidase GII precursor, putative, expressed |
| Os.7991.1.S1_at | AB003194.1 | 7.06508 | 0.4278058 | Endochitinase A precursor, putative, expressed |
| Os.53973.1.S1_at | AK101108.1 | 6.40013 | 1.1122951 | Endoglucanase 1 precursor, putative, expressed |
| Os.28030.1.S1_at | AK104912.1 | 1.79919 | 1.5448543 | Xyloglucan endotransglucosylase/hydrolase protein 15precursor, putative, expressed |
| Os.10166.1.S1_at | AB096140.1 | 5.68145 | 1.5448543 | Endochitinase A precursor, putative, expressed |
| Os.28030.1.S1_s_at | AK104912.1 | 3.33796 | 1.8606407 | Xyloglucan endotransglucosylase/hydrolase protein 15precursor, putative, expressed |
| Os.9759.1.S1_x_at | AK106049.1 | 2.5295 | 1.8606407 | Polygalacturonase family protein, expressed |
| Os.2368.1.S1_at | AF247164.1 | 6.10808 | 2.3550221 | Alpha-expansin 4 precursor, putative, expressed |
| Os.7314.1.S1_at | AK065000.1 | 2.08714 | 2.6108037 | Cellulase containing protein, expressed |
| Os.56129.1.S1_at | AK109306.1 | 1.53432 | 3.0257366 | Pectinesterase inhibitor domain containing protein |
| **Probe Set ID** | **Representative Public ID** | **Fold Change** | **q-value(%)** | **Description** |
| Os.4159.1.S1_at | AF030167.1 | 2.11154 | 3.0257366 | Glucan endo-1,3-beta-glucosidase GII precursor, putative, expressed |
| OsAffx.31336.1.S1_at | 9639.m03339 | 2.10645 | 3.0765608 | Glucan endo-1,3-beta-glucosidase 5 precursor, putative, expressed |
| Os.51106.1.S1_at | AK060686.1 | 3.55277 | 3.0765608 | Endoglucanase 1 precursor, putative, expressed |
| Os.51174.1.S1_at | AK061284.1 | 3.89733 | 3.101945 | Xyloglucan endotransglucosylase/hydrolase protein 28precursor, putative, expressed,Xyloglucan Endotransglucosylase Activity Loosens a Plant Cell Wall. |
| Os.51228.2.A1_at | AK062288.1 | 2.20064 | 3.101945 | proline-rich protein, putative, expressed |
| Os.32182.1.S1_at | AK101962.1 | 1.90462 | 3.101945 | Pectinesterase family protein, expressed |
| Os.2416.1.S1_a_at | AB027428.1 | 1.78042 | 4.3419899 | Glucan endo-1,3-beta-glucosidase, acidic isoform precursor, putative, expressed |
| Os.47330.1.S1_at | AK105110.1 | 2.17676 | 4.8372211 | Pectinesterase, putative, expressed |
| Os.12838.1.S1_at | AK069642.1 | 2.09567 | 4.8372211 | Pectinesterase inhibitor domain containing protein, expressed |
| Os.8203.1.S1_at | AK099480.1 | 3.98321 | 5.8786028 | Proline-rich protein, putative, expressed |
| Os.21574.1.S1_a_at | AK120399.1 | 1.59959 | 5.8786028 | Glucan endo-1,3-beta-glucosidase 7 precursor, putative, expressed |
| OsAffx.31336.1.S1_x_at | 9639.m03339 | 1.87425 | 5.8786028 | Glucan endo-1,3-beta-glucosidase 5 precursor, putative, expressed |
| OsAffx.12459.1.S1_at | 9630.m04087 | 2.50255 | 5.8786028 | Beta-expansin 3 precursor, putative, expressed |
| **Phytohormone related** | | | | |
|  |  |  |  |  |
| Os.11266.1.S1_at | AK103553.1 | 3.78826 | 0 | Auxin-induced protein PCNT115, putative, expressed |
| OsAffx.28989.1.S1_s_at | 9636.m00152 | 2.01364 | 0 | Auxin-induced protein 10A5, putative, expressed |
| Os.21805.1.S1_s_at | CA756790 | 7.66005 | 0 | Gibberellin regulated protein, expressed |
| Os.46435.1.S1_s_at | BE040077 | 4.09372 | 0.8078121 | Gibberellin regulated protein, expressed |
| Os.39652.1.S1_at | CB667155 | 2.22815 | 1.8606407 | Auxin induced protein, putative, expressed |
| Os.11762.1.S2_x_at | AK121506.1 | 1.95683 | 1.8606407 | P-glycoprotein 1, putative, expressed |
| Os.9945.1.S1_at | AK073044.1 | 2.07712 | 2.4099727 | AUX/IAA family protein, expressed |
| Os.48278.2.S1_x_at | AK101780.1 | 1.6323 | 3.101945 | Auxin-regulated protein, putative, expressed |
| Os.31907.1.S1_x_at | AK106518.1 | 2.21684 | 3.101945 | P-glycoprotein, putative, expressed |
| Os.8639.1.S1_at | AK063247.1 | 2.59794 | 4.3419899 | Auxin-induced protein TGSAUR12, putative, expressed |
| Os.37213.1.S1_at | AK060076.1 | 1.56852 | 4.3419899 | Auxin responsive protein, expressed |
| Os.51408.1.S1_at | AK062728.1 | 1.5243 | 4.3419899 | Auxin responsive protein |
| Os.33170.1.S1_x_at | AK101504.1 | 2.47927 | 5.8786028 | Auxin efflux carrier protein, putative, expressed |
| OsAffx.15981.1.S1_at | 9634.m04702 | 1.61563 | 5.8786028 | Brassinosteroid-regulated protein BRU1 precursor, putative, expressed |
| **Transcription factor** | | | | |
|  |  |  |  |  |
| Os.35343.1.A1_at | AK067922.1 | 12.9456 | 0 | NAC-domain containing protein 21/22, putative, expressed |
| Os.16301.1.S1_at | BI802275 | 3.67513 | 0 | Histone-like transcription factor and archaeal histone family protein, expressed |
| Os.26488.1.S1_at | AK068593.1 | 3.04961 | 0 | Helix-loop-helix DNA-binding domain containing protein, expressed |
| Os.56365.1.S1_at | AK109929.1 | 3.12183 | 0 | bZIP transcription factor family protein, expressed |
| Os.28406.1.S1_at | AK106057.1 | 2.8497 | 0 | AP2 domain containing protein, expressed |
| Os.39872.1.A1_s_at | CF321377 | 4.96292 | 0 | No apical meristem, putative, expressed |
| OsAffx.31559.1.S1_at | 9640.m00213 | 2.28731 | 0 | No apical meristem protein, expressed |
|  |  |  |  |  |
| **Probe Set ID** | **Representative Public ID** | **Fold Change** | **q-value(%)** | **Description** |
| Os.50585.1.S1_at | AK121953.1 | 4.36723 | 0 | No apical meristem protein, expressed |
| Os.16301.1.S1_at | BI802275 | 3.67513 | 0 | Histone-like transcription factor and archaeal histone family protein, expressed |
| Os.2365.1.S1_at | AY206864.1 | 2.42657 | 0 | SlHDL2, putative, expressed |
| Os.5733.1.A1_s_at | BI305452 | 3.12825 | 0.4278058 | Zinc finger, C2H2 type family protein, expressed |
| Os.2066.2.S1_x_at | NM_188429.1 | 1.67696 | 0.4278058 | TCP family transcription factor containing protein, expressed |
| Os.9336.1.S1_at | AK060276.1 | 1.84317 | 0.4278058 | Myb-related protein Zm38, putative, expressed |
| Os.18595.1.A1_at | AK068606.1 | 3.18267 | 0.4278058 | NAC-domain containing protein 18, putative, expressed |
| Os.37618.1.S1_at | AK102692.1 | 6.53261 | 0.4278058 | Homeobox-leucine zipper protein HAT3, putative, expressed |
| Os.37618.1.S1_at | AK102692.1 | 6.53261 | 0.4278058 | Homeobox-leucine zipper protein HAT3, putative, expressed |
| Os.56943.1.S1_at | AK110611.1 | 1.90334 | 0.8078121 | No apical meristem, putative, expressed |
| Os.49956.1.S1_at | AK119431.1 | 1.62372 | 1.1122951 | C2H2 zinc-finger protein, putative, expressed |
| Os.38278.1.S1_at | AK070845.1 | 3.99802 | 1.1122951 | B3 DNA binding domain containing protein, expressed |
| Os.26538.1.S1_at | AY466471.1 | 2.15555 | 1.5448543 | Transcription factor RF2b, putative, expressed |
| Os.33336.1.S1_at | AK100944.1 | 2.61712 | 1.5448543 | bZIP transcription factor family protein, expressed |
| Os.55684.1.S1_at | AK108553.1 | 8.53621 | 1.5448543 | bZIP transcription factor family protein, expressed |
| Os.18448.1.S1_at | AK107297.1 | 1.84436 | 1.8606407 | myb-like DNA-binding domain, SHAQKYF class family protein, expressed |
| Os.55986.1.S1_x_at | AK109091.1 | 2.1961 | 1.8606407 | Dof domain, zinc finger family protein, expressed |
| Os.51204.1.S1_at | AK061568.1 | 2.65175 | 2.3550221 | TCP family transcription factor containing protein, expressed |
| Os.26054.1.S1_s_at | BM038927 | 1.72506 | 2.3550221 | Helix-loop-helix DNA-binding domain containing protein, expressed |
| Os.49501.1.A1_at | AK064240.1 | 2.02534 | 2.4099727 | TCP family transcription factor containing protein, expressed |
| Os.54213.1.S1_at | AK102562.1 | 1.82691 | 2.6108037 | Transcription factor RF2b, putative |
| Os.4901.1.S1_at | AU181981 | 2.15989 | 2.6108037 | Myb-like DNA-binding domain containing protein, expressed |
| Os.56356.1.S1_at | AK109915.1 | 7.03089 | 2.6108037 | Helix-loop-helix DNA-binding domain containing protein, expressed |
| OsAffx.12587.1.S1_s_at | 9630.m05041 | 2.45814 | 2.6108037 | bHLH protein, putative, expressed |
| Os.51150.1.S1_at | AK061163.1 | 3.22833 | 2.6108037 | AP2 domain-containing transcription factor family protein, putative, expressed |
| Os.9917.1.S1_s_at | AU089792 | 1.78434 | 3.0257366 | Transcription factor S-II family protein, expressed |
| Os.18192.1.S1_at | CB618799 | 1.50832 | 3.0257366 | bZIP family transcription factor, putative, expressed |
| Os.15992.1.S1_at | AK107524.1 | 1.89488 | 3.0257366 | Dof domain, zinc finger family protein, expressed |
| OsAffx.30765.1.S1_at | 9639.m00047 | 1.6756 | 3.0765608 | Myb-like DNA-binding domain, SHAQKYF class family protein, expressed |
| Os.9971.1.S1_s_at | BX898176 | 2.12232 | 3.0765608 | Myb-like DNA-binding domain containing protein, expressed |
| Os.408.1.S1_a_at | AY398581.1 | 2.37495 | 3.0765608 | Myb-like DNA-binding domain containing protein, expressed |
| Os.22375.1.S1_x_at | AK059468.1 | 3.64355 | 3.0765608 | MYB1, putative, expressed |
| Os.23028.1.S1_s_at | AK106987.1 | 1.50031 | 3.0765608 | AP2 domain containing protein, expressed |
| Os.51078.1.S1_at | AK106041.1 | 0.35538 | 3.0765608 | AP2 domain containing protein, expressed |
| Os.50556.1.S1_at | AK121826.1 | 1.75514 | 3.101945 | Zinc finger, C2H2 type family protein, expressed |
| Os.18448.1.S1_s_at | AK107297.1 | 1.79104 | 3.101945 | Myb-like DNA-binding domain, SHAQKYF class family protein, expressed |
| Os.52592.1.S1_at | AK068228.1 | 3.57622 | 3.101945 | Helix-loop-helix DNA-binding domain containing protein, expressed |
| Os.54660.1.S1_at | AK106398.1 | 1.68999 | 3.101945 | CCAAT-binding transcription factor subunit B family protein, expressed |
| **Probe Set ID** | **Representative Public ID** | **Fold Change** | **q-value(%)** | **Description** |
| Os.14318.1.S1_at | AK059041.1 | 4.87989 | 3.101945 | bHLH transcription factor GBOF-1, putative, expressed |
| Os.51150.1.S1_s_at | AK061163.1 | 4.87315 | 3.101945 | AP2 domain-containing transcription factor family protein, putative, expressed |
| Os.10115.1.S1_at | AK065915.1 | 1.60059 | 4.3419899 | Myb-related protein Hv33, putative, expressed |
| Os.10316.1.S1_at | AK106333.1 | 1.80356 | 4.3419899 | Helix-loop-helix DNA-binding domain containing protein, expressed |
| Os.51967.1.S1_at | AK064429.1 | 1.96475 | 4.3419899 | bZIP transcription factor family protein |
| OsAffx.14261.4.S1_x_at | 9631.m02180 | 2.14818 | 4.8372211 | Transcription factor RF2b, putative, expressed |
| OsAffx.14273.1.S1_at | 9632.m04339 | 1.95276 | 4.8372211 | Heavy-metal-associated domain-containing protein, putative, expressed |
| Os.53575.1.S1_at | AK073378.1 | 1.779 | 4.8372211 | bHLH transcription factor bHLH033, putative, expressed |
| Os.19843.1.S1_at | AK058570.1 | 2.12807 | 5.8786028 | TCP1 protein, putative, expressed |
| Os.17286.1.S1_at | CB647995 | 1.63018 | 5.8786028 | NAC domain transcription factor, putative, expressed |
| Os.7751.1.S1_at | AK119183.1 | 1.69167 | 5.8786028 | Helix-loop-helix DNA-binding domain containing protein, expressed |
| Os.46563.1.S1_a_at | AK063669.1 | 1.69499 | 5.8786028 | Helix-loop-helix DNA-binding domain containing protein, expressed |
| OsAffx.2611.1.S1_at | 9630.m01391 | 2.62964 | 5.8786028 | bZIP transcription factor family protein, expressed |
| **Cytoskeleton or cytosjeleton associated** | | | | |
|  |  |  |  |  |
| Os.11740.1.S1_at | AK103340.1 | 1.54 | 2.6108037 | Actin-97, putative, expressed |
| Os.49435.1.S1_at | AK121803.1 | 1.66571 | 2.6108037 | 65kD microtubule associated protein, putative, expressed |
| Os.3422.1.S1_at | AK063598.1 | 1.51787 | 3.0257366 | Actin-3, putative, expressed |
| Os.18675.1.S1_at | CR290536 | 1.58567 | 3.0765608 | Actin-1, putative, expressed |
| Os.49634.1.S1_x_at | AK120226.1 | 0.55065 | 4.8372211 | Dynein light chain type 1 family protein, expressed |
| Os.12198.1.S1_a_at | AK102553.1 | 1.93441 | 5.8786028 | Microtubule-associated protein MAP65-1a, putative, expressed |
| **Cell cycle related** | | | | |
|  |  |  |  |  |
| Os.46852.1.S1_at | AK107529.1 | 3.49617 | 0 | Cyclin, N-terminal domain containing protein, expressed |
| OsAffx.32170.1.S1_at | 9640.m04277 | 6.74637 | 0.4278058 | P21 protein, putative, expressed |
| Os.8306.1.S1_at | AK070478.1 | 1.9541 | 3.0257366 | Cyclin, N-terminal domain containing protein, expressed |
| Os.52202.1.S1_at | AK065579.1 | 1.96125 | 3.0765608 | Rad9 family protein, expressed |
| Os.51953.1.S1_at | AK064396.1 | 1.55656 | 5.8786028 | Cyclin, N-terminal domain containing protein, expressed |
| Os.18607.1.S1_at | AK063940.1 | 1.90522 | 5.8786028 | Cyclin delta-2, putative, expressed |
| **Secretory pathway** | | | | |
|  |  |  |  |  |
| Os.18952.1.S1_at | AK100316.1 | 2.36477 | 0 | Syntaxin 132, putative, expressed |
| Os.28649.1.S1_at | AK111255.1 | 0.40109 | 0.4278058 | Zn-finger in Ran binding protein and others containing protein, expressed |
| Os.17918.1.S1_at | AK061425.1 | 3.7956 | 0.4278058 | 33 kDa secretory protein, putative, expressed |
| Os.3933.1.S1_a_at | AK103200.1 | 1.55669 | 1.5448543 | Trafficking protein particle complex subunit 3, putative, expressed |
| Os.51147.1.S1_at | AK061148.1 | 2.16289 | 1.5448543 | 33 kDa secretory protein, putative, expressed |
| Os.52791.1.S1_at | AK069270.1 | 2.10904 | 1.8606407 | Dynamin-related protein 1C, putative, expressed |
| Os.46450.1.S1_at | AK100816.1 | 2.30662 | 2.3550221 | Secretory protein, putative, expressed |
|  |  |  |  |  |
| **Probe Set ID** | **Representative Public ID** | **Fold Change** | **q-value(%)** | **Description** |
| OsAffx.30676.2.S1_x_at | 9638.m03079 | 2.30115 | 2.3550221 | Secretory protein, putative |
| Os.13565.1.A1_s_at | CR284436 | 1.64775 | 3.0765608 | Reticulon family protein, putative, expressed |
| Os.54339.1.S1_at | AK103492.1 | 1.76797 | 3.0765608 | Exocyst subunit EXO70 family protein, putative, expressed |
| OsAffx.15295.1.S1_x_at | 9634.m00653 | 1.66663 | 4.3419899 | Syntaxin 132, putative, expressed |
| Os.12383.1.S1_at | AK102713.1 | 1.68133 | 5.8786028 | Ras-related protein Rab11C, putative, expressed |
| **Transposon/retrotransposon protein / RNA silencing pathway genes/possible genome stress induced genes** | | | | |
|  |  |  |  |  |
| Os.46273.1.S1_at | NM_194846.1 | 2.10119 | 0 | Retrotransposon protein, putative, Ty1-copia subclass |
| OsAffx.8864.1.S1_at | NM_194007.1 | 11.9376 | 0 | Retrotransposon protein, putative, Ty1-copia subclass |
| OsAffx.28651.1.S1_x_at | 9635.m02806 | 3.10055 | 0 | Piwi domain containing protein, expressed |
| Os.7338.1.S1_at | AK069685.1 | 5.49833 | 0 | Piwi domain containing protein, expressed |
| OsAffx.12575.1.S1_at | 9630.m04931 | 3.60111 | 0 | RNA-directed RNA polymerase 2, putative, expressed |
| Os.24032.2.S1_at | AK073006.1 | 1.99127 | 0.4278058 | Transposon protein, putative, CACTA, En/Spm sub-class |
| Os.13051.1.S1_at | AB079873.1 | 5.44807 | 1.1122951 | Meiotic recombination protein DMC1 homolog, putative, expressed |
| Os.14561.1.S1_at | AK101363.1 | 1.92045 | 1.1122951 | Type IIB DNA topoisomerase family protein, expressed |
| Os.18126.1.S1_at | C97874 | 4.04738 | 2.4099727 | Transposon protein, putative, unclassified, expressed |
| Os.54641.1.S1_at | AK106346.1 | 3.292 | 3.0257366 | Transposon protein, putative, unclassified, expressed |
| Os.54579.1.S1_x_at | AK105945.1 | 2.51206 | 3.0257366 | Transposon protein, putative, CACTA, En/Spm sub-class, expressed |
| Os.8113.1.S1_at | AK103199.1 | 3.28992 | 3.0257366 | Transposon protein, putative, CACTA, En/Spm sub-class, expressed |
| OsAffx.9699.1.S1_at | NM_189633.1 | 1.54479 | 3.0257366 | Retrotransposon protein, putative, LINE subclass |
| Os.11839.1.S1_s_at | BQ908269 | 1.8469 | 3.0257366 | RuvB-like 1, putative, expressed |
| Os.55691.1.S1_at | AK108572.1 | 1.63286 | 3.0257366 | Complex 1 protein containing protein, expressed,Yeast Chromatin Assembly Complex 1 Protein Excludes Nonacetylatable Forms of Histone H4 from Chromatin and the Nucleus |
| Os.10497.1.S1_s_at | AK105956.1 | 2.57946 | 3.0765608 | Transposon protein, putative, CACTA, En/Spm sub-class, expressed |
| Os.3406.1.S1_at | AB004865.1 | 2.71587 | 3.101945 | Transposon protein, putative, unclassified, expressed |
| Os.54579.1.S1_s_at | AK105945.1 | 2.38623 | 3.101945 | Transposon protein, putative, CACTA, En/Spm sub-class, expressed |
| Os.24718.1.S1_at | AK100570.1 | 1.74392 | 3.101945 | DNA polymerase V family protein, expressed |
| Os.5562.1.S1_at | AB007452.1 | 2.23122 | 4.3419899 | Transposon protein, putative, unclassified, expressed |
| OsAffx.28901.3.S1_at | 9631.m05179 | 2.49619 | 4.3419899 | Transposon protein, putative, Mutator sub-class, expressed |
| Os.11832.1.S1_at | AK101096.1 | 1.88081 | 4.3419899 | Transposon protein, putative, CACTA, En/Spm sub-class, expressed |
| Os.6042.1.S1_at | AU166923 | 2.00967 | 4.3419899 | Transposon protein, putative, CACTA, En/Spm sub-class, expressed |
| Os.13991.1.A1_at | CB630751 | 1.54859 | 4.3419899 | Hypothetical protein , retrotransposon protein, putative, Ty3-gypsy subclass |
| Os.50333.1.S1_at | AK120766.1 | 1.746 | 4.3419899 | Piwi domain containing protein, expressed |
| Os.51851.1.S1_at | AK105369.1 | 1.89073 | 4.8372211 | Transposon protein, putative, CACTA, En/Spm sub-class, expressed |
| Os.27140.1.S1_a_at | AK068125.1 | 3.80648 | 4.8372211 | Retrotransposon protein, putative, Ty1-copia subclass, expressed |
| Os.6203.1.S1_at | AK101485.1 | 2.04036 | 4.8372211 | DNA repair ATPase, putative, expressed |
|  |  |  |  |  |
| **Probe Set ID** | **Representative Public ID** | **Fold Change** | **q-value(%)** | **Description** |
| Os.50472.2.S1_x_at | AK121327.1 | 1.69829 | 5.8786028 | Transposable element protein, putative, Retrotrans_gag |
| Os.46580.1.S1_at | AK062808.1 | 1.74656 | 5.8786028 | Retrotransposon protein, putative, Ty3-gypsy subclass, expressed |
| Os.11065.1.A1_at | CR285534 | 2.26966 | 5.8786028 | Retrotransposon protein, putative, Ty3-gypsy subclass, expressed |
| Os.7466.1.S1_at | AK063836.1 | 1.58525 | 5.8786028 | Single-strand binding protein family protein, expressed |
| **Protein synthesis** | | | | |
|  |  |  |  |  |
| Os.11498.1.S1_at | AK121755.1 | 1.7351 | 0.4278058 | 60S ribosomal protein L18a, putative, expressed |
| Os.446.1.S1_at | AK103139.1 | 4.17256 | 0.4278058 | Hypothetical protein , Eukaryotic translation initiation factor 6, putative, expressed |
| Os.24658.1.S1_at | AK061110.1 | 3.20975 | 0.4278058 | Methionine aminopeptidase 2, putative, expressed |
| Os.9405.1.S1_at | AK120520.1 | 1.84496 | 1.1122951 | 40S ribosomal protein S11, putative, expressed |
| Os.18206.1.S1_at | AK062929.1 | 1.81075 | 1.8606407 | 60S ribosomal protein L30, putative, expressed |
| Os.5668.2.S1_x_at | AK107780.1 | 2.1737 | 1.8606407 | tRNA pseudouridine synthase family protein, putative, expressed |
| Os.12590.1.S1_at | AK070995.1 | 1.70608 | 2.4099727 | 60S ribosomal protein L13-2, putative, expressed , Superoxide dismutase, chloroplast, putative, expressed |
| Os.12280.1.S1_x_at | AK060433.1 | 1.51945 | 2.4099727 | 60S acidic ribosomal protein P1, putative, expressed |
| Os.6267.1.S1_at | AK121702.1 | 1.73196 | 2.6108037 | 60S ribosomal protein L44, putative, expressed |
| Os.51511.1.S1_at | AK062985.1 | 1.67246 | 2.6108037 | 50S ribosomal protein L20, putative, expressed |
| Os.23431.1.A1_at | AK121443.1 | 1.56792 | 3.0257366 | Ribosomal protein L24 containing protein, expressed |
| Os.9188.1.S1_x_at | AK058490.1 | 1.52703 | 3.0257366 | 60S ribosomal protein L7 containing protein, expressed |
| Os.9188.1.S1_at | AK058490.1 | 1.60384 | 3.0257366 | 60S ribosomal protein L7 containing protein, expressed |
| Os.37611.2.S1_x_at | CR282531 | 1.70152 | 3.0257366 | 60S acidic ribosomal protein P2A, putative, expressed |
| Os.9193.1.S1_at | L36313.1 | 1.60154 | 3.0257366 | 40S ribosomal protein S16, putative, expressed |
| OsAffx.12818.1.S1_s_at | 9631.m01007 | 1.76412 | 3.0257366 | Mitochondrial ribosomal protein L51/S25/CI-B8 family protein, putative, expressed |
| Os.53888.1.S1_at | AK100630.1 | 1.7812 | 3.0765608 | Ribosomal protein L7Ae/L30e/S12e/Gadd45 family protein, putative, expressed |
| Os.6282.1.S1_at | AK062099.1 | 1.62566 | 3.0765608 | Ribosomal L28e protein family protein, expressed |
| Os.9268.2.S1_at | AK059679.1 | 1.84209 | 3.0765608 | 60S ribosomal protein L38, putative, expressed |
| Os.37611.1.S1_at | AK058815.1 | 1.53274 | 3.0765608 | 60S acidic ribosomal protein P2A, putative, expressed |
| Os.16825.1.S1_at | AK063732.1 | 1.76842 | 3.0765608 | 40S ribosomal protein S2, putative, expressed |
| Os.25094.1.A1_at | AK070231.1 | 1.63081 | 3.101945 | Ribosomal protein L7/L12 C-terminal domain containing protein, expressed |
| Os.9941.1.S1_at | AK072488.1 | 1.50728 | 3.101945 | 60S ribosomal protein L17, putative, expressed |
| OsAffx.5287.1.S1_s_at | 9635.m01033 | 1.77947 | 3.101945 | 40S ribosomal protein S2, putative, expressed |
| Os.13629.1.S1_at | BI796766 | 1.63392 | 3.101945 | mitochondrial ribosomal protein L51/S25/CI-B8 family protein, putative, expressed |
| Os.12596.1.S1_at | AK067896.1 | 1.61714 | 4.3419899 | 60S ribosomal protein L6, putative, expressed |
| Os.1082.1.S1_a_at | AK105054.1 | 1.59611 | 4.3419899 | 60S ribosomal protein L29, putative |
| Os.25811.1.S1_at | AU108592 | 2.24912 | 4.3419899 | 60S acidic ribosomal protein P0, putative, expressed |
| Os.9946.1.S1_at | AK073778.1 | 1.66964 | 4.3419899 | 50S ribosomal protein L3-2, chloroplast precursor, putative, expressed |
|  |  |  |  |  |
| **Probe Set ID** | **Representative Public ID** | **Fold Change** | **q-value(%)** | **Description** |
| Os.57446.1.S1_x_at | D29724.1 | 1.52905 | 4.3419899 | Peptide chain release factor 2, putative, expressed , 60S ribosomal protein L38, putative, expressed |
| Os.9075.1.S1_at | AK102675.1 | 1.55633 | 4.8372211 | 40S ribosomal protein S23, putative, expressed ,PHD finger protein, putative, expressed |
| OsAffx.12705.1.S1_at | 9631.m00095 | 1.50851 | 4.8372211 | 40S ribosomal protein S17, putative, expressed |
| Os.4622.1.S1_at | AK062943.1 | 1.59193 | 4.8372211 | 40S ribosomal protein S15a, putative, expressed |
| Os.33544.1.S1_at | AK100177.1 | 1.90235 | 4.8372211 | Aminoacyl-tRNA synthetase family, putative, expressed ,bHLH transcription factor, putative, expressed |
| Os.16649.1.S1_at | AK067243.1 | 1.99471 | 5.8786028 | Ribosomal protein L1 containing protein, expressed |
| Os.17308.1.S1_at | AK106546.1 | 1.50355 | 5.8786028 | Initiator tRNA phosphoribosyl transferase family protein, expressed |
| **Genes with no annotated function** | | | | |
|  |  |  |  |  |
| Os.9498.1.S1_at | AK064358.1 | 1.65809 | 0 | Expressed protein |
| Os.8034.1.S1_at | AK107956.1 | 1.83149 | 0 | Expressed protein |
| Os.50023.1.S1_at | AK119712.1 | 2.21081 | 0 | Expressed protein |
| Os.52927.1.S1_at | AK069962.1 | 2.23785 | 0 | Expressed protein |
| Os.12700.1.S1_at | AK103655.1 | 2.26116 | 0 | Expressed protein |
| Os.52285.1.S1_at | AK066082.1 | 2.28356 | 0 | Expressed protein |
| Os.11920.1.S1_s_at | AK102569.1 | 2.59596 | 0 | Expressed protein |
| Os.6288.1.S1_at | AK106356.1 | 2.87021 | 0 | Expressed protein |
| Os.10862.1.S1_at | CF340108 | 3.3094 | 0 | Expressed protein |
| Os.18455.1.S1_at | AK108146.1 | 3.43422 | 0 | Expressed protein |
| Os.10245.1.S1_at | AK108406.1 | 3.60301 | 0 | Expressed protein |
| Os.37860.1.S1_at | AK063683.1 | 3.60471 | 0 | Expressed protein |
| OsAffx.27508.7.S1_s_at | AK110901.1 | 3.64696 | 0 | Expressed protein |
| OsAffx.7530.1.S1_s_at | 9640.m00831 | 3.66046 | 0 | Expressed protein |
| Os.36687.1.A1_at | AK062883.1 | 3.71325 | 0 | Expressed protein |
| Os.50118.1.S1_at | AK100822.1 | 4.4061 | 0 | Expressed protein |
| Os.52280.1.S1_at | AK066054.1 | 4.71304 | 0 | Expressed protein |
| Os.15780.1.S1_at | AK110943.1 | 4.76337 | 0 | Expressed protein |
| Os.17758.1.A1_at | AK108811.1 | 4.84442 | 0 | Expressed protein |
| Os.12988.1.S1_at | AK106819.1 | 5.12736 | 0 | Expressed protein |
| Os.51864.1.S1_at | AK064113.1 | 5.93802 | 0 | Expressed protein |
| Os.31171.1.S1_at | AK107734.1 | 6.49956 | 0 | Expressed protein |
| Os.47910.1.S1_at | AK105313.1 | 6.52982 | 0 | Expressed protein |
| Os.50180.1.S1_at | AK119998.1 | 7.63075 | 0 | Expressed protein |
| Os.10682.1.S1_at | AK109215.1 | 8.002 | 0 | Expressed protein |
| Os.17985.1.S1_s_at | C96822 | 8.1906 | 0 | Expressed protein |
| OsAffx.16847.1.S1_s_at | 9636.m00528 | 8.55708 | 0 | Expressed protein |
| Os.45916.1.S1_s_at | AU082531 | 8.69817 | 0 | Expressed protein |
| **Probe Set ID** | **Representative Public ID** | **Fold Change** | **q-value(%)** | **Description** |
| Os.4618.1.S1_at | AK062310.1 | 10.8685 | 0 | Expressed protein |
| Os.7108.1.S1_at | AK109082.1 | 11.2946 | 0 | Expressed protein |
| Os.11513.1.S1_at | AK107485.1 | 16.965 | 0 | Expressed protein |
| OsAffx.26739.1.S1_s_at | 9633.m00439 | 24.2118 | 0 | Expressed protein |
| Os.15440.1.S1_at | AK067932.1 | 2.45418 | 0 |  |
| Os.47923.2.A1_at | AK062615.1 | 2.50315 | 0 |  |
| Os.54623.1.S2_at | AK106277.1 | 2.55236 | 0 |  |
| Os.28435.1.S1_a_at | AK067086.1 | 2.80943 | 0 |  |
| Os.15983.1.S1_at | CA765179 | 3.02849 | 0 |  |
| Os.10636.2.A1_x_at | NM_185608.1 | 3.22054 | 0 |  |
| Os.4952.1.S1_at | AK070970.1 | 3.62662 | 0 |  |
| Os.37644.1.S1_at | AK107857.1 | 6.00072 | 0 |  |
| OsAffx.16847.1.A1_at | 9636.m00528 | 8.74814 | 0 |  |
| OsAffx.30103.2.S1_s_at | 9637.m02741 | 20.2274 | 0 |  |
| Os.57298.1.S1_at | AK111259.1 | 1.97297 | 0 |  |
| Os.46584.1.S1_at | AK061597.1 | 3.86745 | 0 | Hypothetical protein |
| Os.46728.1.S1_at | CB000629 | 4.94856 | 0 | Hypothetical protein |
| Os.1408.1.S1_at | AK102840.1 | 1.73595 | 0.4278058 | Expressed protein |
| Os.11899.1.S1_at | AK069349.1 | 1.85709 | 0.4278058 | Expressed protein |
| Os.18461.1.S1_at | AK069754.1 | 2.60919 | 0.4278058 | Expressed protein |
| Os.8503.1.S1_at | AK062563.1 | 3.76781 | 0.4278058 | Expressed protein |
| Os.56000.1.S1_at | AK109108.1 | 3.86113 | 0.4278058 | Expressed protein |
| Os.9962.1.S1_s_at | AK060341.1 | 14.9184 | 0.4278058 | Expressed protein |
| Os.28435.4.S1_x_at | AK066303.1 | 1.87284 | 0.4278058 |  |
| Os.54551.1.S1_at | AK105580.1 | 2.04673 | 0.4278058 |  |
| Os.57207.1.S1_s_at | AK111139.1 | 1.72375 | 0.8078121 | Expressed protein ,TPR Domain containing protein, expressed |
| Os.45916.1.S1_x_at | AU082531 | 12.8212 | 0.8078121 | Expressed protein |
| Os.49480.1.S1_at | AK110531.1 | 2.30031 | 0.8078121 | hypothetical protein |
| Os.12798.1.S1_at | NM_190392.1 | 2.0749 | 1.1122951 | Expressed protein |
| Os.6417.1.S1_at | AK062834.1 | 19.1839 | 1.1122951 | Expressed protein |
| Os.50472.2.S1_s_at | AK121327.1 | 2.16944 | 1.1122951 |  |
| Os.10636.1.A1_at | AK068486.1 | 3.69122 | 1.1122951 |  |
| OsAffx.30103.2.S1_at | 9637.m02741 | 6.31769 | 1.1122951 |  |
| Os.16479.1.S1_at | AK121337.1 | 1.59813 | 1.1122951 | Uncharacterised protein family containing protein, expressed |
| Os.49381.1.S1_at | AK101110.1 | 1.8331 | 1.5448543 | Expressed protein |
| Os.50023.1.S1_x_at | AK119712.1 | 2.06067 | 1.5448543 | Expressed protein |
| Os.22968.1.A1_at | CR285033 | 2.3246 | 1.5448543 | Expressed protein |
| Os.51855.1.S1_at | AK064092.1 | 2.37839 | 1.5448543 | Expressed protein |
| Os.12999.1.S1_at | AK063036.1 | 2.55713 | 1.5448543 | Expressed protein |
| Os.50349.2.S1_at | AK107892.1 | 2.63396 | 1.5448543 | Expressed protein |
|  |  |  |  |  |
| **Probe Set ID** | **Representative Public ID** | **Fold Change** | **q-value(%)** | **Description** |
| Os.16839.1.S1_at | CB620847 | 1.50192 | 1.5448543 |  |
| Os.47879.1.A1_at | AK111367.1 | 4.00264 | 1.5448543 |  |
| Os.53575.1.S1_s_at | AK073378.1 | 4.36823 | 1.5448543 |  |
| Os.39994.1.S1_at | CF319063 | 3.07832 | 1.5448543 | Hypothetical protein |
| Os.14289.1.S1_at | AK107139.1 | 2.61349 | 1.8606407 | Expressed protein , epoxide hydrolase, putative, expressed |
| Os.12921.1.S1_at | AU222951 | 1.56547 | 1.8606407 | Expressed protein |
| OsAffx.12740.1.S1_s_at | 9631.m00475 | 1.86778 | 1.8606407 | Expressed protein |
| Os.27509.1.S1_at | CB642018 | 2.69987 | 1.8606407 | Expressed protein |
| Os.35704.1.S1_at | AK066200.1 | 3.42794 | 1.8606407 | Expressed protein |
| Os.7213.1.S1_at | AK064760.1 | 1.78461 | 1.8606407 |  |
| Os.47700.1.A1_at | CR286943 | 1.86044 | 1.8606407 |  |
| Os.13565.1.A1_at | CR284436 | 1.98024 | 1.8606407 |  |
| Os.26888.1.A1_at | CB626658 | 1.98203 | 1.8606407 |  |
| Os.4914.1.S1_s_at | AU182100 | 2.6543 | 1.8606407 |  |
| Os.8113.2.S1_x_at | NM_189545.1 | 2.84645 | 1.8606407 |  |
| Os.52745.1.S1_at | AK069029.1 | 3.70514 | 1.8606407 |  |
| Os.25564.1.S1_at | AK101915.1 | 3.76334 | 1.8606407 |  |
| Os.56789.1.S1_at | AK110418.1 | 2.57875 | 2.3550221 | Expressed protein |
| Os.40421.1.S1_at | AK102492.1 | 7.48905 | 2.3550221 | Expressed protein |
| Os.49671.1.S1_at | AK101945.1 | 8.63674 | 2.3550221 | Expressed protein |
| Os.10409.1.A1_at | AK064062.1 | 1.68583 | 2.3550221 |  |
| Os.18501.1.S1_at | AK111015.1 | 2.14697 | 2.3550221 |  |
| Os.28044.1.A1_at | BU672803 | 4.78944 | 2.3550221 |  |
| Os.27542.1.A1_at | AK062794.1 | 1.57063 | 2.3550221 | Hypothetical protein |
| Os.55681.1.S1_x_at | AK108548.1 | 1.54012 | 2.4099727 | Expressed protein |
| Os.52147.1.S1_at | AK065205.1 | 1.84104 | 2.4099727 | Expressed protein |
| Os.11225.1.S1_at | AK068696.1 | 3.11683 | 2.4099727 | Expressed protein |
| Os.48741.1.S1_at | CR288096 | 2.23582 | 2.4099727 |  |
| Os.16859.1.S1_at | AK062755.1 | 2.29776 | 2.4099727 |  |
| Os.12111.1.S1_at | AK099618.1 | 2.40763 | 2.4099727 |  |
| Os.8252.1.S1_at | AK102373.1 | 3.29971 | 2.6108037 | Expressed protein |
| Os.7555.1.S1_at | AK107813.1 | 1.55538 | 2.6108037 | Expressed protein |
| Os.55116.1.S1_at | AK107513.1 | 1.64419 | 2.6108037 | Expressed protein |
| Os.8736.1.S1_a_at | AK121371.1 | 1.79091 | 2.6108037 | Expressed protein |
| Os.16164.1.S1_at | AK062309.1 | 1.87621 | 2.6108037 | Expressed protein |
| Os.13757.1.S1_at | AK060374.1 | 1.8788 | 2.6108037 | Expressed protein |
| Os.10797.1.S1_at | AK120063.1 | 1.89415 | 2.6108037 | Expressed protein |
| Os.31338.1.S1_at | AK107502.1 | 2.459 | 2.6108037 | Expressed protein |
| Os.23125.1.S1_at | AK103709.1 | 2.6337 | 2.6108037 | Expressed protein |
| Os.2426.1.A1_at | CA759372 | 3.12895 | 2.6108037 | Expressed protein |
| Os.35583.1.S1_at | AK066764.1 | 3.28472 | 2.6108037 | Expressed protein |
| **Probe Set ID** | **Representative Public ID** | **Fold Change** | **q-value(%)** | **Description** |
| Os.50771.1.S1_at | AK058439.1 | 4.05876 | 2.6108037 | Expressed protein |
| Os.50846.1.S1_s_at | AK058956.1 | 10.423 | 2.6108037 | Expressed protein |
| Os.27778.2.S1_at | AK068030.1 | 1.58506 | 2.6108037 |  |
| Os.6259.1.S1_at | AK064274.1 | 1.71754 | 2.6108037 |  |
| Os.29822.1.S1_at | AK109802.1 | 1.81018 | 2.6108037 |  |
| Os.28435.5.S1_x_at | AK099678.1 | 1.91962 | 2.6108037 |  |
| Os.52101.1.S1_at | AK064720.1 | 2.03629 | 2.6108037 |  |
| Os.54497.1.S1_at | AK105358.1 | 1.66047 | 2.6108037 | Hypothetical protein |
| Os.50419.1.S1_x_at | AK072780.1 | 2.30796 | 2.6108037 | Uncharacterized plant-specific domain TIGR01570 family protein, expressed |
| Os.51888.1.S1_at | AK064196.1 | 1.52553 | 3.0257366 | Expressed protein |
| Os.38074.1.S1_s_at | CF328719 | 1.57053 | 3.0257366 | Expressed protein |
| Os.7488.1.S1_s_at | BI796568 | 1.59446 | 3.0257366 | Expressed protein |
| Os.5378.2.S1_x_at | AK110670.1 | 1.63246 | 3.0257366 | Expressed protein |
| Os.10857.1.S1_at | AK063945.1 | 1.79824 | 3.0257366 | Expressed protein |
| Os.27197.2.A1_at | AK067842.1 | 1.97848 | 3.0257366 | Expressed protein |
| Os.31233.2.S1_at | AK121188.1 | 2.05363 | 3.0257366 | Expressed protein |
| Os.56189.1.S1_at | AK109579.1 | 2.31565 | 3.0257366 | Expressed protein |
| Os.52058.1.S1_at | AK064637.1 | 3.41564 | 3.0257366 | Expressed protein |
| OsAffx.22588.1.S1_at | 9635.m04969 | 3.52202 | 3.0257366 | Expressed protein |
| Os.4980.1.S1_at | AK059286.1 | 4.21696 | 3.0257366 | Expressed protein |
| Os.55317.1.S1_at | AK107856.1 | 1.60936 | 3.0257366 |  |
| Os.42953.1.S1_x_at | NM_189475.1 | 1.64489 | 3.0257366 |  |
| Os.9411.1.S1_at | AU091954 | 1.89403 | 3.0257366 |  |
| Os.17722.1.S1_at | AK058883.1 | 2.03105 | 3.0257366 |  |
| Os.12606.1.S1_at | AK071496.1 | 0.27006 | 3.0257366 |  |
| OsAffx.32262.1.S1_x_at | X15901.1 | 0.49467 | 3.0257366 |  |
| Os.9836.1.S1_at | AK059202.1 | 5.24632 | 3.0257366 | Hypothetical protein |
| Os.23932.1.A1_at | AK108082.1 | 2.36327 | 3.0765608 | Expressed protein ,Rf1 protein, mitochondrial precursor, putative, expressed |
| Os.27169.1.S1_s_at | CB645166 | 1.55362 | 3.0765608 | Expressed protein |
| Os.12353.1.S1_at | AK120721.1 | 1.60513 | 3.0765608 | Expressed protein |
| Os.7699.1.S1_at | AK120490.1 | 1.63266 | 3.0765608 | Expressed protein |
| Os.55094.1.S1_at | AK107479.1 | 1.7448 | 3.0765608 | Expressed protein |
| Os.57009.1.S1_at | AK110737.1 | 2.05459 | 3.0765608 | Expressed protein |
| Os.52377.1.S1_s_at | AK066722.1 | 2.12247 | 3.0765608 | Expressed protein |
| Os.9551.1.S1_at | AK101753.1 | 2.28724 | 3.0765608 | Expressed protein |
| Os.5875.1.S1_at | AK110593.1 | 2.69555 | 3.0765608 | Expressed protein |
| Os.38995.1.S1_at | CF291340 | 1.8088 | 3.0765608 |  |
| Os.26380.1.S1_at | AK101933.1 | 1.81372 | 3.0765608 |  |
| Os.9956.1.S1_at | AU082529 | 2.56487 | 3.0765608 |  |
| Os.31771.2.S1_x_at | AK106475.1 | 2.93116 | 3.0765608 |  |
| Os.7317.2.S1_at | AK105463.1 | 5.69813 | 3.0765608 |  |
| **Probe Set ID** | **Representative Public ID** | **Fold Change** | **q-value(%)** | **Description** |
| Os.27569.1.S2_at | AK121706.1 | 0.38924 | 3.0765608 |  |
| Os.37668.1.S1_at | CB625667 | 0.54852 | 3.0765608 |  |
| OsAffx.23371.1.S1_x_at | 9629.m02360 | 1.59344 | 3.101945 | Expressed protein |
| Os.8987.1.S1_at | AK121328.1 | 1.63515 | 3.101945 | Expressed protein |
| OsAffx.30108.1.S1_at | 9637.m02780 | 1.83621 | 3.101945 | Expressed protein |
| Os.50862.1.S1_x_at | AK059055.1 | 1.94739 | 3.101945 | Expressed protein |
| Os.54502.1.S1_at | AK105452.1 | 2.19544 | 3.101945 | Expressed protein |
| OsAffx.12645.1.S1_s_at | 9630.m05446 | 2.67592 | 3.101945 | Expressed protein |
| Os.12412.1.S1_at | AK069678.1 | 3.09784 | 3.101945 | Expressed protein |
| Os.11897.1.S1_at | AK065702.1 | 4.49956 | 3.101945 | Expressed protein |
| Os.53609.1.S1_s_at | AK073568.1 | 6.34985 | 3.101945 | Expressed protein |
| Os.8352.1.S1_at | AK102406.1 | 1.72814 | 3.101945 | Expressed protein, hypothetical protein |
| Os.14771.1.S1_at | AK061209.1 | 1.50962 | 3.101945 |  |
| Os.37339.1.S1_at | AK059163.1 | 1.51738 | 3.101945 |  |
| Os.8596.1.S1_at | AK103286.1 | 1.54491 | 3.101945 |  |
| Os.55382.1.S1_at | AK107983.1 | 1.63591 | 3.101945 |  |
| Os.37362.1.S1_at | AK058997.1 | 1.65981 | 3.101945 |  |
| Os.49347.1.S1_at | AK102157.1 | 1.74264 | 3.101945 |  |
| Os.47362.1.A1_at | BX899638 | 1.89311 | 3.101945 |  |
| Os.11221.1.S1_at | AK071131.1 | 1.91542 | 3.101945 |  |
| Os.18164.1.S1_at | C98103 | 2.04399 | 3.101945 |  |
| Os.14100.1.S1_at | BI804947 | 2.04453 | 3.101945 |  |
| Os.18311.1.S1_at | AK070697.1 | 2.31158 | 3.101945 |  |
| Os.11303.1.S1_at | AK107822.1 | 2.34582 | 3.101945 |  |
| Os.20157.1.A1_at | CR288123 | 2.95274 | 3.101945 |  |
| Os.9283.1.S1_at | AK121139.1 | 1.84621 | 3.101945 | Hypothetical protein |
| Os.27232.1.S1_at | AK107951.1 | 2.12068 | 3.101945 | Uncharacterized plant-specific domain TIGR01615 family protein, expressed |
| Os.49192.1.S1_at | AK071152.1 | 1.6485 | 4.3419899 | Expressed protein ,Vignain precursor, putative |
| Os.8252.1.S1_s_at | AK102373.1 | 3.31468 | 4.3419899 | Expressed protein |
| Os.56288.1.S1_at | AK109773.1 | 1.50839 | 4.3419899 | Expressed protein |
| Os.10189.1.S1_at | AK121756.1 | 1.53267 | 4.3419899 | Expressed protein |
| OsAffx.29192.1.S1_at | 9636.m01415 | 1.53581 | 4.3419899 | Expressed protein |
| Os.22793.1.S1_at | AK063374.1 | 1.54053 | 4.3419899 | Expressed protein |
| Os.28031.1.S1_at | AK107710.1 | 1.69905 | 4.3419899 | Expressed protein |
| Os.54078.1.S1_at | AK101737.1 | 1.7005 | 4.3419899 | Expressed protein |
| Os.50862.1.S1_at | AK059055.1 | 1.71517 | 4.3419899 | Expressed protein |
| Os.24493.1.A1_at | CB634459 | 1.72195 | 4.3419899 | Expressed protein |
| Os.49596.1.S1_at | AK102580.1 | 1.75359 | 4.3419899 | Expressed protein |
| Os.24554.1.A1_at | AK067397.1 | 1.80723 | 4.3419899 | Expressed protein |
| Os.7515.1.S1_s_at | AK070601.1 | 1.83393 | 4.3419899 | Expressed protein |
| **Probe Set ID** | **Representative Public ID** | **Fold Change** | **q-value(%)** | **Description** |
| Os.53554.1.S1_at | AK073259.1 | 2.18641 | 4.3419899 | Expressed protein |
| OsAffx.12022.1.S1_s_at | 9630.m01254 | 2.23156 | 4.3419899 | Expressed protein |
| Os.50118.2.S1_x_at | AY224472.1 | 2.26931 | 4.3419899 | Expressed protein |
| Os.49679.1.S1_at | AK101663.1 | 2.52655 | 4.3419899 | Expressed protein |
| Os.55272.1.S1_at | AK107776.1 | 2.77723 | 4.3419899 | Expressed protein |
| Os.9524.1.S2_at | AK073036.1 | 2.97879 | 4.3419899 | Expressed protein |
| Os.51649.1.S1_at | AK063263.1 | 3.04997 | 4.3419899 | Expressed protein |
| Os.50843.1.S1_at | AK058938.1 | 6.6593 | 4.3419899 | Expressed protein |
| Os.10310.1.S1_at | AK064825.1 | 1.53235 | 4.3419899 |  |
| Os.6435.1.S1_at | AK103593.1 | 1.63522 | 4.3419899 |  |
| Os.9155.1.S1_at | AK062922.1 | 1.68785 | 4.3419899 |  |
| Os.27861.1.S2_a_at | AK068557.1 | 1.70158 | 4.3419899 |  |
| Os.18124.1.S1_x_at | C97872 | 1.73176 | 4.3419899 |  |
| Os.9840.1.S1_at | AK106038.1 | 2.03502 | 4.3419899 |  |
| Os.12282.1.S1_a_at | AK068026.1 | 2.4794 | 4.3419899 |  |
| Os.7575.1.S1_at | AU101420 | 3.18779 | 4.3419899 |  |
| Os.45982.1.A1_x_at | CB651281 | 2.2271 | 4.3419899 | Hypothetical protein |
| Os.6561.1.A1_at | AU165189 | 2.51917 | 4.3419899 | Hypothetical protein |
| Os.6231.1.S1_at | AK108274.1 | 1.63981 | 4.8372211 | Expressed protein |
| Os.49548.1.S1_at | AK107906.1 | 1.66014 | 4.8372211 | Expressed protein |
| Os.7588.1.S1_at | CR289174 | 1.73233 | 4.8372211 | Expressed protein |
| Os.49112.1.S1_at | AK108180.1 | 1.74599 | 4.8372211 | Expressed protein |
| Os.32434.1.S1_at | AK104975.1 | 1.82885 | 4.8372211 | Expressed protein |
| OsAffx.11145.1.S1_s_at | 9629.m02065 | 1.83055 | 4.8372211 | Expressed protein |
| Os.14119.1.S1_x_at | AK105615.1 | 1.83889 | 4.8372211 | Expressed protein |
| Os.14077.1.S1_at | AK108341.1 | 1.92936 | 4.8372211 | Expressed protein |
| Os.18512.1.S1_at | AK072498.1 | 1.96143 | 4.8372211 | Expressed protein |
| Os.51245.1.S1_at | AK062326.1 | 1.97603 | 4.8372211 | Expressed protein |
| OsAffx.14410.1.S1_s_at | 9632.m05269 | 2.12455 | 4.8372211 | Expressed protein |
| Os.53873.1.S1_x_at | AK100544.1 | 2.24597 | 4.8372211 | Expressed protein |
| Os.27974.1.S1_at | AK121555.1 | 2.38095 | 4.8372211 | Expressed protein |
| Os.31675.1.S1_at | AK106937.1 | 2.42411 | 4.8372211 | Expressed protein |
| Os.15580.1.S1_at | AK062353.1 | 2.4767 | 4.8372211 | Expressed protein |
| OsAffx.27508.94.S1_s_at | AK062277.1 | 2.87476 | 4.8372211 | Expressed protein |
| Os.55342.1.S1_at | AK107904.1 | 2.94439 | 4.8372211 | Expressed protein |
| OsAffx.11813.1.S1_at | NM_190079.1 | 3.79889 | 4.8372211 | Expressed protein |
| Os.53184.1.S1_at | AK071256.1 | 1.54978 | 4.8372211 |  |
| Os.54244.1.S1_at | AK102755.1 | 1.58702 | 4.8372211 |  |
| **Probe Set ID** | **Representative Public ID** | **Fold Change** | **q-value(%)** | **Description** |
| Os.27176.1.S1_at | AK101182.1 | 1.7471 | 4.8372211 |  |
| Os.20292.1.S1_at | AK071680.1 | 1.75024 | 4.8372211 |  |
| OsAffx.8778.1.S1_at | XM_353960.1 | 1.78235 | 4.8372211 |  |
| Os.7733.1.S2_at | AK064467.1 | 1.78445 | 4.8372211 |  |
| OsAffx.16737.1.S1_at | AK067579.1 | 1.89126 | 4.8372211 |  |
| Os.7736.1.S1_at | AU101939 | 2.03353 | 4.8372211 |  |
| Os.10255.1.S1_s_at | AK060977.1 | 2.42998 | 4.8372211 |  |
| Os.10038.1.S1_s_at | AU082861 | 0.37388 | 4.8372211 | Hypothetical protein ,Expressed protein |
| Os.50013.1.S1_at | AK119634.1 | 1.69233 | 4.8372211 | Uncharacterized plant-specific domain TIGR01615 family protein, expressed |
| Os.54373.1.S1_at | AK103727.1 | 1.51152 | 5.8786028 | Expressed protein |
| Os.15671.1.A1_at | AK058919.1 | 1.52642 | 5.8786028 | Expressed protein |
| Os.14243.1.S1_at | AK070104.1 | 1.53107 | 5.8786028 | Expressed protein |
| Os.18510.1.S1_at | AK105436.1 | 1.53203 | 5.8786028 | Expressed protein |
| Os.33971.1.S1_at | AK073330.1 | 1.53687 | 5.8786028 | Expressed protein |
| Os.9280.1.S1_a_at | AK102872.1 | 1.59384 | 5.8786028 | Expressed protein |
| Os.22122.1.S1_at | AB079874.1 | 1.68105 | 5.8786028 | Expressed protein |
| Os.5450.1.S1_at | AK059387.1 | 1.69979 | 5.8786028 | Expressed protein |
| Os.6224.2.S1_at | AK107787.1 | 1.71314 | 5.8786028 | Expressed protein |
| Os.15725.1.S1_at | AK100733.1 | 1.75837 | 5.8786028 | Expressed protein |
| Os.27394.1.A1_x_at | CB642698 | 1.80229 | 5.8786028 | Expressed protein |
| Os.14119.2.S1_x_at | NM_197732.1 | 1.89853 | 5.8786028 | Expressed protein |
| Os.57301.1.S1_at | AK111266.1 | 2.0125 | 5.8786028 | Expressed protein |
| Os.50366.1.S1_x_at | AK120891.1 | 2.10636 | 5.8786028 | Expressed protein |
| Os.36549.1.S1_at | AK063135.1 | 2.1101 | 5.8786028 | Expressed protein |
| Os.34913.1.S1_at | AK069648.1 | 2.23035 | 5.8786028 | Expressed protein |
| Os.30696.1.S1_at | AK108464.1 | 2.44294 | 5.8786028 | Expressed protein |
| Os.46941.1.S1_s_at | AK100361.1 | 2.55899 | 5.8786028 | Expressed protein |
| Os.50018.1.S1_at | AK119672.1 | 2.91449 | 5.8786028 | Expressed protein |
| Os.56185.1.S1_at | AK109572.1 | 3.12285 | 5.8786028 | Expressed protein |
| Os.41519.1.S1_at | NM_192389.1 | 3.14893 | 5.8786028 | Expressed protein |
| Os.42815.1.S1_at | D23155 | 1.50409 | 5.8786028 |  |
| Os.28301.1.S2_at | CB647177 | 1.59137 | 5.8786028 |  |
| Os.54352.1.S1_at | AK103584.1 | 1.63179 | 5.8786028 |  |
| Os.52026.2.S1_at | AK064559.1 | 1.63354 | 5.8786028 |  |
| Os.49358.1.S1_at | AK059462.1 | 1.63927 | 5.8786028 |  |
| Os.12342.1.S2_at | CB668460 | 1.68422 | 5.8786028 |  |
| Os.16158.1.S1_at | AK103120.1 | 1.7545 | 5.8786028 |  |
| Os.38052.1.S1_at | AK070584.1 | 1.76877 | 5.8786028 |  |
| Os.22058.1.S1_at | BM038035 | 1.78143 | 5.8786028 |  |
| Os.56136.1.S1_at | AK109320.1 | 1.81847 | 5.8786028 |  |
| Os.56325.1.S1_at | AK109857.1 | 2.40404 | 5.8786028 |  |
| Os.11559.1.S1_at | AK071392.1 | 2.96052 | 5.8786028 |  |
| **Probe Set ID** | **Representative Public ID** | **Fold Change** | **q-value(%)** | **Description** |
| Os.30054.1.S1_at | AK109381.2 | 3.43854 | 5.8786028 |  |
| Os.50382.1.S1_at | AK065818.1 | 5.64456 | 5.8786028 |  |
| Os.54810.1.A1_at | AK106813.1 | 2.02445 | 5.8786028 | Hypothetical protein ,Coatomer delta subunit, putative, expressed |
| Os.11520.1.S1_at | AK062517.1 | 5.51738 | 5.8786028 | Hypothetical protein |
| Os.39982.1.S1_at | CF312224 | 1.8362 | 5.8786028 | Uncharacterized ACR, COG1565 family protein, expressed |
| **Unclassified** | | | | |
|  |  |  |  |  |
| Os.37921.2.S1_at | AK071014.1 | 3.26167 | 0 | Zinc finger family protein, putative, expressed |
| Os.51900.1.S1_at | AK064220.1 | 4.24316 | 0 | Von Willebrand factor type A domain containing protein, expressed |
| Os.27799.1.S1_at | AK105614.1 | 2.73163 | 0 | Vacuolar sorting receptor 7 precursor, putative, expressed |
| Os.9042.1.S1_at | AY576526.1 | 1.60799 | 0 | Ras-related protein ARA-3, putative, expressed |
| Os.52717.1.S1_at | AK068902.1 | 5.84336 | 0 | Pumilio-family RNA binding repeat containing protein, expressed |
| Os.7393.1.S1_at | AK073121.1 | 3.02215 | 0 | Plastocyanin-like domain containing protein, expressed |
| Os.28139.1.S1_at | AK060655.1 | 8.39601 | 0 | Osmotin-like protein precursor, putative, expressed |
| Os.28462.1.S1_s_at | AF051369.1 | 4.24477 | 0 | Nonspecific lipid-transfer protein 5 precursor, putative, expressed |
| OsAffx.30533.1.S1_s_at | NM_196352.1 | 1.79112 | 0 | Mitochondrial carrier protein, expressed |
| Os.52847.1.S1_at | AK069582.1 | 3.6253 | 0 | IBR domain containing protein, expressed |
| Os.2321.1.S1_at | AF141879.1 | 4.37082 | 0 | Germin-like protein subfamily 1 member 11 precursor, putative, expressed |
| Os.51561.1.S1_at | AK063071.1 | 28.4882 | 0 | GDSL-motif lipase/hydrolase, putative |
| Os.12340.1.S1_at | AK058429.1 | 2.60865 | 0 | GDSL-like Lipase/Acylhydrolase family protein, expressed |
| Os.5270.1.S1_at | AK061147.1 | 10.9642 | 0 | GDSL-like Lipase/Acylhydrolase family protein, expressed |
| Os.28229.2.S1_x_at | NM_191862.1 | 2.95826 | 0 | GDSL-like Lipase/Acylhydrolase family protein |
| Os.54871.1.S1_at | AK106964.1 | 5.42911 | 0 | Fasciclin-like arabinogalactan protein 7 precursor, putative, expressed |
| Os.33625.4.S1_at | Z34270.1 | 3.09315 | 0 | Esterase PIR7B, putative, expressed |
| Os.22715.1.S1_at | AK103046.1 | 2.20832 | 0 | DHHC zinc finger domain containing protein, expressed |
| Os.9592.1.S1_at | AK121884.1 | 2.29598 | 0 | COBRA-like protein 2 precursor, putative, expressed |
| Os.10884.1.S1_at | AK070731.1 | 3.78584 | 0 | ATPase 2, putative, expressed |
| Os.56290.1.S1_at | AK109777.1 | 3.6796 | 0 | AMP-binding enzyme family protein, expressed |
| Os.54874.1.S1_at | AK106970.1 | 8.71392 | 0 | GEX1, putative, expressed |
| OsAffx.3365.1.S1_s_at | 9631.m02900 | 2.61415 | 0 | Small nuclear ribonucleoprotein G, putative, expressed |
| OsAffx.24153.1.S1_s_at | 9630.m00720 | 2.23596 | 0.4278058 | VQ motif family protein, expressed |
| Os.14770.1.S1_at | AK060649.1 | 7.15234 | 0.4278058 | RNA recognition motif family protein, expressed |
| Os.18433.1.S1_at | BM421610 | 1.95723 | 0.4278058 | NLI interacting factor-like phosphatase family protein, expressed |
| Os.11252.1.S1_at | AK102380.1 | 2.08241 | 0.4278058 | Heavy metal-associated domain containing protein, expressed |
| Os.7727.1.S1_at | AY580163.1 | 3.14655 | 0.4278058 | GDSL-like Lipase/Acylhydrolase family protein, expressed |
| Os.34478.1.S1_at | AK071235.1 | 3.09851 | 0.4278058 | DnaJ domain containing protein, expressed |
| Os.18433.1.S1_s_at | BM421610 | 1.92383 | 0.8078121 | NLI interacting factor-like phosphatase family protein, expressed |
| **Probe Set ID** | **Representative Public ID** | **Fold Change** | **q-value(%)** | **Description** |
| Os.23169.1.S1_at | AK060611.1 | 1.70955 | 0.8078121 | Brix domain containing protein 2, putative, expressed,a role in ribosome biogenesis and rRNA binding,involved in the multistep process of the assembly of the large ribosomal subunit |
| Os.28229.1.S1_at | AK070946.1 | 6.06028 | 1.1122951 | GDSL-like Lipase/Acylhydrolase family protein, expressed |
| OsAffx.12765.1.S1_x_at | 9631.m00676 | 2.46815 | 1.1122951 | DNA-binding protein, putative, expressed |
| OsAffx.11639.1.S1_at | 9629.m05677 | 1.72313 | 1.5448543 | YOR3513c, putative |
| Os.48459.1.A1_at | CR283190 | 2.22557 | 1.5448543 | SNELIPTRC, putative, expressed |
| Os.6098.1.A1_at | AK065416.1 | 2.13246 | 1.5448543 | PrMC3, putative, expressed |
| OsAffx.31072.1.S1_at | 9639.m01888 | 3.88955 | 1.5448543 | O-methyltransferase family protein, expressed |
| Os.26710.1.S1_at | AK111327.1 | 3.74403 | 1.5448543 | non-phototropic hypocotyl protein, putative |
| Os.36709.1.S1_x_at | AK062840.1 | 1.54725 | 1.5448543 | Heavy metal-associated domain containing protein, expressed |
| Os.27483.1.S1_at | AK059812.1 | 3.91503 | 1.5448543 | Germin-like protein subfamily 1 member 7 precursor, putative, expressed |
| Os.8707.2.A1_at | AK059168.1 | 2.12516 | 1.5448543 | GDSL-motif lipase/hydrolase family protein, putative, expressed |
| OsAffx.27459.2.S1_s_at | 9634.m00419 | 2.05568 | 1.5448543 | Early nodulin 93 ENOD93 protein, expressed |
| Os.10839.1.S1_at | AK106069.1 | 1.65801 | 1.5448543 | DNA-binding protein, putative, expressed |
| OsAffx.28902.1.S1_s_at | 9635.m04484 | 1.66155 | 1.8606407 | Thymidylate kinase family protein, expressed |
| Os.54176.1.S1_at | AK102366.1 | 2.63333 | 1.8606407 | Pumilio-family RNA binding repeat containing protein, expressed |
| Os.9194.1.S1_at | AK070419.1 | 5.61917 | 1.8606407 | Phosphate-induced protein 1, putative, expressed |
| Os.18395.1.S1_s_at | AK062516.1 | 2.26133 | 1.8606407 | GAST1 protein precursor, putative, expressed |
| Os.183.1.S1_at | L36094.1 | 1.70826 | 1.8606407 | EF-1 guanine nucleotide exchange domain containing protein, expressed |
| Os.46749.1.S1_x_at | AK121859.1 | 1.80547 | 1.8606407 | Annexin family protein, expressed |
| Os.8383.1.S1_at | AK059663.1 | 2.10204 | 2.3550221 | T-complex protein 1, beta subunit, putative, expressed |
| Os.36953.1.S1_at | AK062302.1 | 2.04314 | 2.3550221 | Pollen-specific kinase partner protein, putative, expressed |
| Os.12221.1.S1_at | U57639.1 | 2.99586 | 2.3550221 | Plasma membrane associated protein, putative, expressed |
| Os.8666.1.S1_at | AK060243.1 | 44.2987 | 2.3550221 | GDSL-like Lipase/Acylhydrolase family protein, expressed |
| OsAffx.2326.1.S1_x_at | NM_191141.1 | 1.79007 | 2.4099727 | Transferrin receptor-like dimerisation domain containing protein, expressed |
| Os.7354.1.S1_at | AK108435.1 | 2.27941 | 2.4099727 | Mitochondrial import inner membrane translocase subunit Tim17 family protein, expressed |
| Os.33892.1.S1_at | AK063969.1 | 1.73327 | 2.4099727 | Lariat debranching enzyme, C-terminal domain containing protein, expressed |
| Os.18019.1.S1_at | AK111905.1 | 2.37011 | 2.6108037 | Transducin family protein, putative, expressed |
| OsAffx.7024.1.S1_at | 9639.m00609 | 2.36631 | 2.6108037 | SNELIPTRC, putative, expressed |
| Os.9314.1.S1_at | AK066740.1 | 1.55814 | 2.6108037 | RNA recognition motif family protein, expressed |
| Os.34020.2.S1_at | CB678541 | 2.5469 | 2.6108037 | Peptide-N4-asparagine amidase A, putative |
| Os.55040.1.S1_at | AK107360.1 | 0.40205 | 2.6108037 | Integral membrane protein DUF6 containing protein, expressed |
| Os.46650.1.S1_at | CR279516 | 2.06033 | 2.6108037 | Hydrolase, alpha/beta fold family protein, expressed |
| Os.962.1.S1_at | AK105015.2 | 3.85721 | 2.6108037 | heavy metal-associated domain containing protein, expressed |
| Os.8707.3.S1_x_at | AK103825.1 | 3.25413 | 2.6108037 | GDSL-motif lipase/hydrolase family protein, putative, expressed |
| **Probe Set ID** | **Representative Public ID** | **Fold Change** | **q-value(%)** | **Description** |
| Os.38638.1.S1_at | AB018376.1 | 3.4487 | 2.6108037 | Early nodulin 93, putative, expressed |
| Os.39913.1.A1_at | CF320837 | 2.29011 | 2.6108037 | C2 domain containing protein, expressed |
| Os.54351.1.S1_at | AK103556.1 | 2.02574 | 2.6108037 | C1-like domain containing protein, expressed |
| OsAffx.6196.1.S1_at | 9637.m00546 | 1.78984 | 2.6108037 | 3' exoribonuclease family, domain 2 containing protein, expressed |
| Os.33686.1.S1_at | AK099501.1 | 2.10064 | 2.6108037 | Ribonucleoprotein, putative, expressed |
| Os.8719.1.A1_s_at | BX928968 | 2.57516 | 3.0257366 | Viral A-type inclusion protein repeat containing protein, expressed |
| OsAffx.12755.1.S1_at | 9631.m00578 | 2.83451 | 3.0257366 | Viral A-type inclusion protein repeat containing protein, expressed |
| Os.11699.1.S1_at | AK109357.2 | 1.59284 | 3.0257366 | T-complex protein 1, eta subunit, putative, expressed |
| Os.10453.1.S1_at | AK067114.1 | 1.65588 | 3.0257366 | T-complex protein 1, epsilon subunit, putative, expressed |
| Os.30812.1.S1_at | AK108287.1 | 2.29648 | 3.0257366 | SNF7 family protein, expressed,vacuolar protein sorting (VPS) genes |
| Os.21902.1.S1_at | AK072474.1 | 2.52556 | 3.0257366 | Remorin, C-terminal region family protein, expressed |
| Os.8225.1.S1_at | AK058507.1 | 1.54308 | 3.0257366 | Pollen-specific protein SF3, putative, expressed |
| Os.37571.1.S1_at | AK063639.1 | 1.8809 | 3.0257366 | Plastocyanin-like domain containing protein, expressed , Expressed protein |
| Os.14564.1.S1_at | AK109337.2 | 1.90263 | 3.0257366 | Peptide-N4-asparagine amidase A, putative, expressed |
| Os.6262.1.S1_at | AK101352.1 | 2.29072 | 3.0257366 | Nuclear transport factor 2 domain containing protein, expressed |
| Os.5025.1.S1_at | AK108100.1 | 2.40342 | 3.0257366 | Nitrate-induced NOI protein, expressed |
| Os.27379.1.S1_at | AK069659.1 | 5.19533 | 3.0257366 | Male sterility protein, expressed |
| Os.8991.1.S1_at | AK062179.1 | 1.64336 | 3.0257366 | Guanine nucleotide-binding protein beta subunit, putative, expressed |
| Os.10533.1.S1_at | AK061118.1 | 4.07633 | 3.0257366 | GDSL-like Lipase/Acylhydrolase family protein, expressed |
| Os.6755.2.S1_at | AK106978.1 | 1.92976 | 3.0257366 | BAG domain-containing protein, putative, expressed |
| OsAffx.18566.1.S1_x_at | NM_195986.1 | 0.40411 | 3.0765608 | NAD, putative ,NADH ubiquinone oxidoreductase, 20 Kd subunit family protein, expressed |
| Os.43682.1.S1_at | CA923293 | 1.75066 | 3.0765608 | Mitochondrial import receptor subunit TOM40 homolog, putative, expressed |
| Os.5985.1.S1_at | AK060664.1 | 3.01136 | 3.0765608 | LysM domain containing protein, expressed |
| Os.7269.1.S1_s_at | CF326526 | 4.14293 | 3.0765608 | Hydrolase, alpha/beta fold family protein, putative, expressed |
| Os.23557.1.A1_at | CA760001 | 2.528 | 3.0765608 | Germin-like protein subfamily 1 member 11 precursor, putative, expressed |
| Os.23398.1.S1_at | AK119282.1 | 2.00269 | 3.0765608 | D-mannose binding lectin family protein, expressed |
| Os.4715.1.S1_at | AK100153.1 | 1.95679 | 3.0765608 | Chemocyanin precursor, putative, expressed |
| Os.5797.1.S1_at | AK101241.1 | 2.91929 | 3.0765608 | Armadillo/beta-catenin-like repeat family protein, expressed |
| Os.26596.1.S1_s_at | NM_195998.1 | 0.58242 | 3.0765608 | NAD, putative , ribosomal protein S15 containing protein , Respiratory-chain NADH dehydrogenase, 49 Kd subunit family protein |
| OsAffx.14384.1.S1_at | 9632.m05127 | 1.73257 | 3.0765608 | BAG domain-containing protein, putative, expressed |
| Os.51369.1.S1_at | AK103542.1 | 2.54058 | 3.101945 | VQ motif family protein, expressed |
| Os.3748.1.S1_at | AK072778.1 | 2.03879 | 3.101945 | Vacuolar ATP synthase subunit E, putative, expressed |
| Os.12326.1.S1_s_at | CA759416 | 1.9191 | 3.101945 | RelA/SpoT containing protein, expressed |
| Os.36684.1.S1_at | AK062889.1 | 4.98563 | 3.101945 | Plastocyanin-like domain containing protein, expressed |
| Os.15008.1.S1_at | AK069844.1 | 2.01443 | 3.101945 | Patellin-5, putative, expressed,membrane traffic |
| Os.18091.1.S1_at | AK058420.1 | 1.69442 | 3.101945 | Mitochondrial glycoprotein, expressed |
| Os.2685.1.S1_at | AK059446.1 | 2.10781 | 3.101945 | Glycine-rich RNA-binding protein 2, mitochondrial precursor, putative, expressed |
| **Probe Set ID** | **Representative Public ID** | **Fold Change** | **q-value(%)** | **Description** |
| Os.13875.1.S1_at | AK060567.1 | 1.98351 | 3.101945 | Glycine cleavage system H protein 1, mitochondrial precursor, putative, expressed |
| Os.52056.1.S1_at | AK064632.1 | 1.91884 | 3.101945 | Exonuclease family protein, expressed |
| Os.27192.1.S1_at | CB647205 | 1.55556 | 3.101945 | Amino acid permease, putative, expressed |
| Os.51184.1.S1_at | AK061404.1 | 2.93259 | 3.101945 | Acid phosphatase, putative, expressed |
| Os.19948.1.S1_at | AK071007.1 | 1.52693 | 3.101945 | Eukaryotic rRNA processing protein EBP2 containing protein, expressed |
| Os.10111.1.S1_at | AK073164.1 | 1.90313 | 3.101945 | Nucleolar protein NOP5, putative, expressed |
| OsAffx.22674.1.S1_at | NM_184875.1 | 2.98859 | 4.3419899 | TPR Domain containing protein |
| Os.18590.1.S1_a_at | AK122176.1 | 1.76991 | 4.3419899 | PINHEAD protein, putative, expressed |
| Os.55640.1.S1_at | AK108467.1 | 1.70571 | 4.3419899 | Mitochondrial prohibitin complex protein 1, putative, expressed |
| Os.30691.1.S1_at | AK108470.1 | 1.5124 | 4.3419899 | Mitochondrial import inner membrane translocase subunit Tim13, putative, expressed , TRANSPARENT TESTA GLABRA 1 protein, putative, expressed |
| Os.47924.1.S1_at | AK100645.1 | 2.49137 | 4.3419899 | Kelch motif family protein, expressed |
| Os.18652.1.S1_at | AK059656.1 | 1.71626 | 4.3419899 | KE2 family protein, expressed |
| Os.6176.1.S1_at | AK073704.1 | 2.06243 | 4.3419899 | Glucosamine-6-phosphate isomerase/6-phosphogluconolactonase family protein, expressed |
| Os.32600.1.S1_at | AK069203.1 | 1.56895 | 4.3419899 | C2 domain-containing protein, putative, expressed |
| Os.16226.1.S1_s_at | BI801996 | 1.7027 | 4.3419899 | 3' exoribonuclease family, domain 2 containing protein, expressed |
| Os.4824.1.A1_s_at | CR280915 | 2.26082 | 4.8372211 | TB2/DP1, HVA22 family protein, expressed |
| Os.7581.1.S1_at | AY288198.2 | 1.97447 | 4.8372211 | RHO protein GDP dissociation inhibitor containing protein, expressed |
| Os.9396.1.S1_at | AK100981.1 | 2.3549 | 4.8372211 | Permease 1, putative, expressed |
| Os.53148.1.S1_at | AK071016.1 | 1.5265 | 4.8372211 | Magnesium-dependent phosphatase-1 family protein, expressed |
| Os.54759.1.S1_at | AK106696.1 | 0.36759 | 4.8372211 | Luminal binding protein 5 precursor, putative ,Luminal binding protein 4 precursor, putative, expressed |
| Os.49735.1.S1_x_at | AK111507.1 | 1.51085 | 4.8372211 | Glutamate-rich WD repeat-containing protein 1, putative, expressed |
| OsAffx.27652.1.S1_x_at | 9634.m01566 | 1.61481 | 4.8372211 | GAST1 protein precursor, putative, expressed |
| Os.5273.2.S1_x_at | CR278699 | 1.59468 | 4.8372211 | DAG protein, chloroplast precursor, putative, expressed |
| Os.8367.1.S1_at | AK068379.1 | 1.85679 | 4.8372211 | AT hook motif family protein, expressed |
| Os.37731.2.S1_at | AU097431 | 1.68074 | 4.8372211 | AFR615Wp, putative, expressed |
| Os.9048.1.S1_at | AK100660.1 | 1.55562 | 4.8372211 | Cleavage and polyadenylation specificity factor, 73 kDa subunit, putative, expressed |
| Os.53192.1.S1_s_at | AK071291.1 | 1.67819 | 4.8372211 | Fibrillarin-2, putative, expressed |
| OsAffx.10304.2.S1_at | 9635.m04550 | 1.55452 | 5.8786028 | Tim44-like domain containing protein, expressed |
| Os.51935.1.S1_at | AK064326.1 | 1.78173 | 5.8786028 | Staphylococcal nuclease homologue family protein, expressed |
| Os.27956.1.S1_at | AK106743.1 | 1.66267 | 5.8786028 | Seed maturation protein, expressed |
| Os.54312.1.S1_at | AK103273.1 | 1.51602 | 5.8786028 | Ribonuclease HI large subunit, putative, expressed |
| Os.6436.1.S1_at | AY046928.1 | 2.17204 | 5.8786028 | Rare lipoprotein A like double-psi beta-barrel containing protein, expressed |
| Os.10032.1.S1_at | AB029509.1 | 2.76496 | 5.8786028 | RAC-like GTP binding protein ARAC7, putative, expressed |
| Os.18687.1.S1_a_at | AK102962.1 | 2.40461 | 5.8786028 | pollen-specific kinase partner protein, putative, expressed |
| **Probe Set ID** | **Representative Public ID** | **Fold Change** | **q-value(%)** | **Description** |
| Os.51195.1.S1_at | AK061513.1 | 2.51517 | 5.8786028 | Nucleoid DNA-binding protein cnd41, putative, expressed |
| Os.10091.1.S1_at | AK061293.1 | 1.60085 | 5.8786028 | long cell-linked locus protein, putative, expressed |
| Os.42475.1.S1_at | AK121828.1 | 1.80244 | 5.8786028 | Fasciclin domain containing protein, expressed |
| Os.19843.1.S1_at | AK058570.1 | 2.12807 | 5.8786028 | TCP1 protein, putative, expressed |
| Os.8783.1.S1_at | AK072392.1 | 1.5049 | 5.8786028 | YT521-B-like family protein, expressed,YT521-B is a ubiquitously expressed nuclear protein that changes alternative splice site usage in a concentration dependent manner |
| Os.6728.2.S1_x_at | AK101143.1 | 1.72487 | 5.8786028 | Nucleolar protein NOP5, putative, expressed |
| Os.6728.1.S1_at | AK099754.1 | 1.76468 | 5.8786028 | Nucleolar protein NOP5, putative, expressed |
