# Supplementary material for: Transcriptome profiling confirmed correlations between symptoms and transcriptional changes in RDV infected rice and revealed nucleolus as a possible target of RDV manipulation
Source: Virol J. 2014 May 6;11:81. doi: 10.1186/1743-422X-11-81 (PMC4032362; doi:10.1186/1743-422X-11-81)
Supplement: Additional file 3: Table S3 — The primers used in this study. [file 1743-422X-11-81-S3.docx]

**Supplemental Table S3. The primers used in this study**

| ***Oryza sativa***  **Genes** | **GB.accession** | **Primer Sequence 5'→3'** | |
| --- | --- | --- | --- |
|  |  |  |  |
| ***RICE* Genes** |  |  | |
|  |  | Primer-F | CTGCTGCTGTTCTTGGGTTCA |
| *OsUBQ11* |  | Primer-R | TCATTATAGTTCTTCCATGCTGCTC |
|  |  | Primer-F | GAATTGAATGAAGCAAAACTGC |
|  | AF247164 | Primer-R | ACCAACCGAAGAAGAACCCA |
|  |  | Primer-F | AGTACCTCTACACCCTCTGCG |
|  | AK059679 | Primer-R | CTGTCTTGATGCTTAAACCTCC |
|  |  | Primer-F | TCTATGGAAAGGGATTAAAGTAGC |
|  | AK061513 | Primer-R | ACACGAGCACAAATGACATCA |
|  |  | Primer-F | TTATCTGCTCGGTGTCCTTACA |
|  | AK062099 | Primer-R | TGCTTACGTTCATAGGCTTTCT |
|  |  | Primer-F | ATCGTGGTTGAGCTGAATGG |
|  | AK062943 | Primer-R | AGGGAAGCAGCCTAGCAGTC |
|  |  | Primer-F | GAACCAGCCCTACGTCATTG |
|  | AK067896 | Primer-R | ATCAACTCGGCATCCACAAC |
|  |  | Primer-F | CCCTCAACGCATCCTACTTC |
|  | AK071291 | Primer-R | ATCCTGTAGCCACCAACGAC |
|  |  | Primer-F | CAACCCTGAACTGTCCCCTAA |
|  | AK099501 | Primer-R | GTCTTGGTCCCCATAATCTTGT |
|  |  | Primer-F | GCTTCGGGTTCTTGAGGGTA |
|  | AK099754 | Primer-R | CAGCCGCAGGATTGTATGTC |
|  |  | Primer-F | CACCGATCCACAGCGTACC |
|  | AK103199 | Primer-R | CCGACAAATCATAACCAAACAA |
|  |  | Primer-F | CATCCTGGGTACAAGACGCT |
|  | AK120766 | Primer-R | CTGGTCCTGTCCCTGTTGTT |
|  |  | Primer-F | AGGATTTGACAACGATTCTGG |
|  | CR282531 | Primer-R | CAAACTTCTCCCTGCCACAA |
|  |  | Primer-F | TGCGGATCAAGAGGACCAAG |
|  | D29724 | Primer-R | GCTACTAATGCGAACGGGAG |
|  |  | Primer-F | GCTTCGAGCTTGTGGACAGT |
|  | U36565 | Primer-R | CGCACCAGCAGATTCCTTAT |
|  |  | Primer-F | CGACCAGTTGTCCTATCTTGC |
|  | AF443600 | Primer-R | CGGCGATGTACTTGATGTTGA |
|  |  | Primer-F | CACGGTAGAGGTCAATGGAAA |
|  | 9631.m02900 | Primer-R | CCAATGCCTCAATCATCACA |
|  |  | Primer-F | GGCACAGTAGTTGATACAGTGATC |
|  | AK069685 | Primer-R | CGTAGTAAACAGGAGGTGGAATAG |
|  |  | Primer-F | GTGTAGACCGGAAATGCAAAC |
|  | AK063247 | Primer-R | AATCCCACGAGCAAGAAGC |
| ***Tobacco* Genes** |  |  | |
|  |  | Primer-F | GGATCTCAAGCGTGGTTATG |
| EF-1-α |  | Primer-R | ATCTCAGCGAACTTGACAGC |
|  |  | Primer-F | TTGTCGGAATGGTCGATGTG |
|  | AB207972 | Primer-R | AATACAGCCTCAGCCGGCAC |
|  |  | Primer-F | TGTCAATCAAGGCCAACTGC |
|  | AM269909 | Primer-R | CCTTCTGCTTCTTCGGCACC |
|  |  | Primer-F | TCAATTGCTGCGGTGGTTGC |
|  | DQ321488 | Primer-R | TCTCTGCGGCTTCTGTCCAG |
|  | M60402 M60403 | Primer-F | TCTTGCATGTTCCATTCCGG |
|  |  | Primer-F | GCCAGCAGCATTGACATAGC |
|  | M60402 M60403 | Primer-R | TTGTTGCCAGCACCATTGAG |
|  |  | Primer-F | TGGTGGTGGTGGTGATGTTC |
|  | AF049353 | Primer-R | ACTGCGGCCATCACCTGTAG |
